# Engineered brain‐targeting exosome for reprogramming immunosuppressive microenvironment of glioblastoma

**DOI:** 10.1002/EXP.20240039

**Published:** 2024-06-26

**Authors:** Jun Yang, Yong Li, Shaoping Jiang, Yuxin Tian, Mengjie Zhang, Shuai Guo, Pengfei Wu, Jianan Li, Lin Xu, Wenpei Li, Yushu Wang, Huile Gao, Yuanyu Huang, Yuhua Weng, Shaobo Ruan

**Affiliations:** ^1^ School of Life Science Advanced Research Institute of Multidisciplinary Science Laboratory of Molecular Medicine and Biotherapy. Beijing Institute of Technology Beijing China; ^2^ Department of Biomedical Engineering Tufts University Medford Massachusetts USA; ^3^ West China School of Pharmacy Sichuan University Chengdu China

**Keywords:** chemo‐resistance, engineered exosomes, glioblastoma, immunosuppressive microenvironment, RNA interference

## Abstract

The immunosuppressive microenvironment of glioblastoma multiforme (GBM) severely impacts the response to various treatments, including systemic chemotherapy. Targeted reprogramming of immunosuppressive GBM microenvironment using RNA interference (RNAi) is largely restricted by poor brain delivery efficiency and targeting specificity. Herein, an acid‐cleavable transferrin (Tf) decorated engineering exosome‐based brain‐targeting delivery system (ACTE) was proposed to efficiently deliver small interference RNA towards transform growth factor‐β (siTGF‐β) and doxorubicin (DOX) to GBM site for combination chemo‐immunotherapy. The siTGF‐β and DOX co‐loaded ACTE, termed as DOX&siTGF‐β@ACTE (Ds@ACTE), is designed to specifically recognize the Tf receptor (TfR) on the blood‐brain barrier (BBB). Subsequently, Ds@ACTE undergoes acid‐responsive detachment of Tf within lysosome of brain capillary endothelial cells, leading to the separation of DOX&siTGF‐β@Exo (Ds@Exo) from the Tf‐TfR complex and enhanced BBB transcytosis. After crossing BBB, the separated Ds@Exo can further target GBM cells via the homing effect. In vivo studies validated that Ds@ACTE significantly downregulated the TGF‐β expression to reprogram the immunosuppressive microenvironment, and thereby reinforce the chemotherapeutic effect of DOX and DOX‐induced anti‐tumor immune response. The effectiveness of this strategy not only can provide thinking for designing a more intelligent brain‐targeting system based on engineered exosomes but also explore an effective treatment regimen for GBM.

## Graphical abstract

An acid‐cleavable Tf‐modified engineering exosome‐based brain‐targeting delivery system (ACTE) was proposed using metabolic glycoengineering and bioorthogonal click chemistry, enabling co‐delivery of siTGF‐β and DOX for GBM treatment. ACTE was featured by higher BBB transcytosis efficiency and hierarchical targeting delivery to GBM cells. Ds@ACTE effectively reprogrammed the immunosuppressive GBM microenvironment, reducing chemoresistance and enhancing anti‐GBM immune responses.

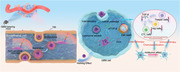

## 1 INTRODUCTION

Glioblastoma multiforme (GBM), the most aggressive and malignant primary intracranial tumor within the central nervous system (CNS), typically confers a poor prognosis upon patients and carries a high risk of recurrence.^[^ ^1^ ^]^ The current standard treatments for GBM encompass a comprehensive approach involving maximum surgical resection, adjuvant radiotherapy, and/or chemotherapy.^[^ ^2^ ^]^ Despite substantial advancements in the field of cancer therapy, the US Food and Drug Administration (FDA) has thus far approved only a very limited number of drugs for GBM treatment.^[^ ^3^ ^]^ Two major factors contributing to this dilemma are the high therapeutic resistance developed by GBM and the presence of the blood‐brain barrier (BBB) that severely restricts the brain entrance of therapeutic drugs.^[^ ^4^ ^]^ Accumulating studies have elucidated the microenvironment of GBM is highly immunosuppressive, which not only can impact the response to cancer immunotherapies but also can impact systemic chemotherapy as well as chemotherapy‐initiated anti‐tumor immune response.^[^ ^5^ ^]^ A plethora of factors have been reported to contribute to the formation of immunosuppressive GBM microenvironment, including the secretion of immunosuppressive cytokines such as transforming growth factor (TGF‐β), interleukin‐10 (IL‐10) and indoleamine‐2,3‐dioxygenase (IDO), the recruitment and differentiation of immunosuppressive cells such as tumor‐associated macrophages (TAMs), regulatory T cells (Tregs), and myeloid‐derived suppressor cells (MDSCs), as well as the expression of immune checkpoint molecules such as programmed death ligand 1 (PD‐L1), programmed death 1 (PD‐1), cytotoxic T lymphocyte antigen 4 (CTLA‐4), and others.^[^ ^6^ ^]^ Among these, TGF‐β, an immunosuppressive cytokine, plays a crucial role in creating an immunosuppressive microenvironment within GBM by suppressing the activation and differentiation of effector T cells, inhibiting the proliferation and cytotoxicity of natural killer cells (NK cells), promoting the differentiation of M2‐phenotype TAMs and Tregs, and hindering the maturation and antigens presentation by dendritic cells (DCs).^[^ ^7^ ^]^ Moreover, TGF‐β promotes the development and maintenance of GBM stem cells, of which several key genes and molecular pathways also contribute to the development of chemotherapy resistance.^[^ ^8^ ^]^ Therefore, downregulating TGF‐β is considered as a promising approach to reverse the immunosuppressive microenvironment and to reduce the chemotherapeutic resistance. Inspired by the potential of RNA interference (RNAi) in regulating gene expression,^[^ ^9^ ^]^ the combination of chemotherapeutic agents with small interfering RNA (siRNA) towards TGF‐β (siTGF‐β) may present a promising treatment regimen for GBM. However, the inherent limitations of siRNA, such as easy degradation by RNases, short blood circulation, poor membrane permeability and lack of target selectivity, and unable to cross BBB, curtail its therapeutic efficacy,^[^ ^10^ ^]^ which also represents similar delivery issues to chemotherapeutic drugs.

The BBB is the major physiological barrier of CNS primarily comprising brain capillary endothelial cells (BCECs) sealed by tight junctions, pericytes, astrocyte end‐feet, and the basement membrane.^[^ ^11^ ^]^ The BBB plays a crucial role in preserving CNS homeostasis through the stringent control of substance transport between the blood and the brain. Additionally, it serves as a protective barrier, preventing the entry of toxins and pathogens into the CNS.^[^ ^12^ ^]^ However, the highly restrictive nature of BBB also presents a barrier to prevent drug delivery to the CNS, posing a formidable challenge for the development of anti‐GBM medications.^[^ ^13^ ^]^ Encouragingly, effective BBB penetration can be achieved by utilizing a brain‐targeted nanoparticle (NP) delivery system designed based on endogenous influx mechanisms.^[^ ^14^ ^]^ These mechanisms primarily include receptor‐mediated transcytosis (RMT), carrier‐mediated transcytosis (CMT), adsorption‐mediated transcytosis (AMT), and cell‐mediated transcytosis, etc.^[^ ^15^ ^]^ Of which, RMT has been extensively investigated for delivering drugs across the BBB due to the expression of abundant receptors on BBB endothelium, such as low‐density lipoprotein receptor (LDLR), transferrin receptor (TfR), insulin receptor, lactoferrin receptor, and scavenger receptor class B type I.^[^ ^16^ ^]^ However, the use of RMT for designing brain‐targeted NPs may encounter a contradiction that low‐affinity ligands require high concentrations to ensure brain‐targeting delivery and effective transcytosis,^[^ ^17^ ^]^ even though they can specifically bind to receptors.^[^ ^18^ ^]^ By contrast, high‐affinity ligands, despite at lower concentrations, are prone to trigger endocytosis by BCECs. However, the strong ligand‐receptor interaction is struggling to dissociate from receptors, resulting in the entrapment of NPs within endo/lysosomes of BCECs and thus lower transcytosis efficiency.^[^ ^19^ ^]^ For instance, transferrin (Tf) exhibits a high affinity for the TfR, and the multivalent strong interactions between Tf and TfR may impede their dissociation, leading to a high proportion of NPs being trapped in endo/lysosomes.^[^ ^17^, ^20^ ^]^ Clark and Davis proposed Tf‐coated gold NPs with acid‐labile linkers, which enabled efficient dissociation of NPs from the Tf‐TfR complex within acidic endo/lysosomes.^[^ ^21^ ^]^ Although this strategy enhanced the brain‐entering number of NPs, the targeting ability is lost after entering the brain parenchyma if the delivery system is intended to target GBM. The introduction of secondary targeting ligands can ensure GBM targeting, whereas it will complicate NPs preparation and make scale‐up manufacturing unfeasible.^[^ ^22^ ^]^ Therefore, reducing BBB entrapment while simultaneously reserving GBM‐targeting ability without additional ligand decoration is necessary for precise treatment.

Exosomes are nanoscale membranous vesicles with diameters ranging from 40 to 150 nm, released by almost all cell types and present in all biological fluids.^[^ ^23^ ^]^ They contain a wealth of bioactive cargoes, including cytosolic and membranous proteins, nucleic acids, lipids, and metabolites, depending on the cell of origin.^[^ ^24^ ^]^ It is now well‐known that exosomes are important mediators of intercellular communication by exchanging bioactive cargoes and thus play crucial roles in regulating physiological conditions and pathological progression.^[^ ^25^ ^]^ Recently, exosomes have gained much interest as drug delivery systems due to their nature drug container property, low immunogenicity, biocompatibility, inherent ability to traverse biological barriers, as well as homing effect.^[^ ^26^ ^]^ Despite of the fact that exosomes were able to cross BBB, their targeting specificity to BBB and transcytosis efficiency remain largely unsatisfied.^[^ ^27^ ^]^ To maximize the brain‐targeting delivery efficiency, engineered exosomes have emerged as a promising alternative.^[^ ^28^ ^]^ The currently developed strategies for engineering exosomes encompass genetic, chemical, physical, biological, as well as microfluidic techniques.^[^ ^29^ ^]^ Among these, chemical modification refers to the attachment of a broader spectrum of natural or synthetic ligands onto the exosome surface through coupling reactions, presenting a popular and flexible engineering approach.^[^ ^30^ ^]^ Recently, metabolic glycoengineering strategies involving the addition of metabolites such as glycans to the culture medium of exosome‐secreting cells have gained significant popularity for introducing modified reactive groups, such as azide groups (N_3_), onto the surface of exosome due to their high specificity and flexibility, simplicity in preparation, mild experimental conditions, and minimal toxicity.^[^ ^31^ ^]^ The targeting molecule of interest can be chemically modified onto the exosome surface through a bioorthogonal reaction with the N_3_ on the surface of the exosome. Therefore, how to rationally engineer exosomes while simultaneously reserving their inherent homing ability is critical to troubleshooting the above‐mentioned delivery challenges.^[^ ^32^ ^]^

**FIGURE 1:**
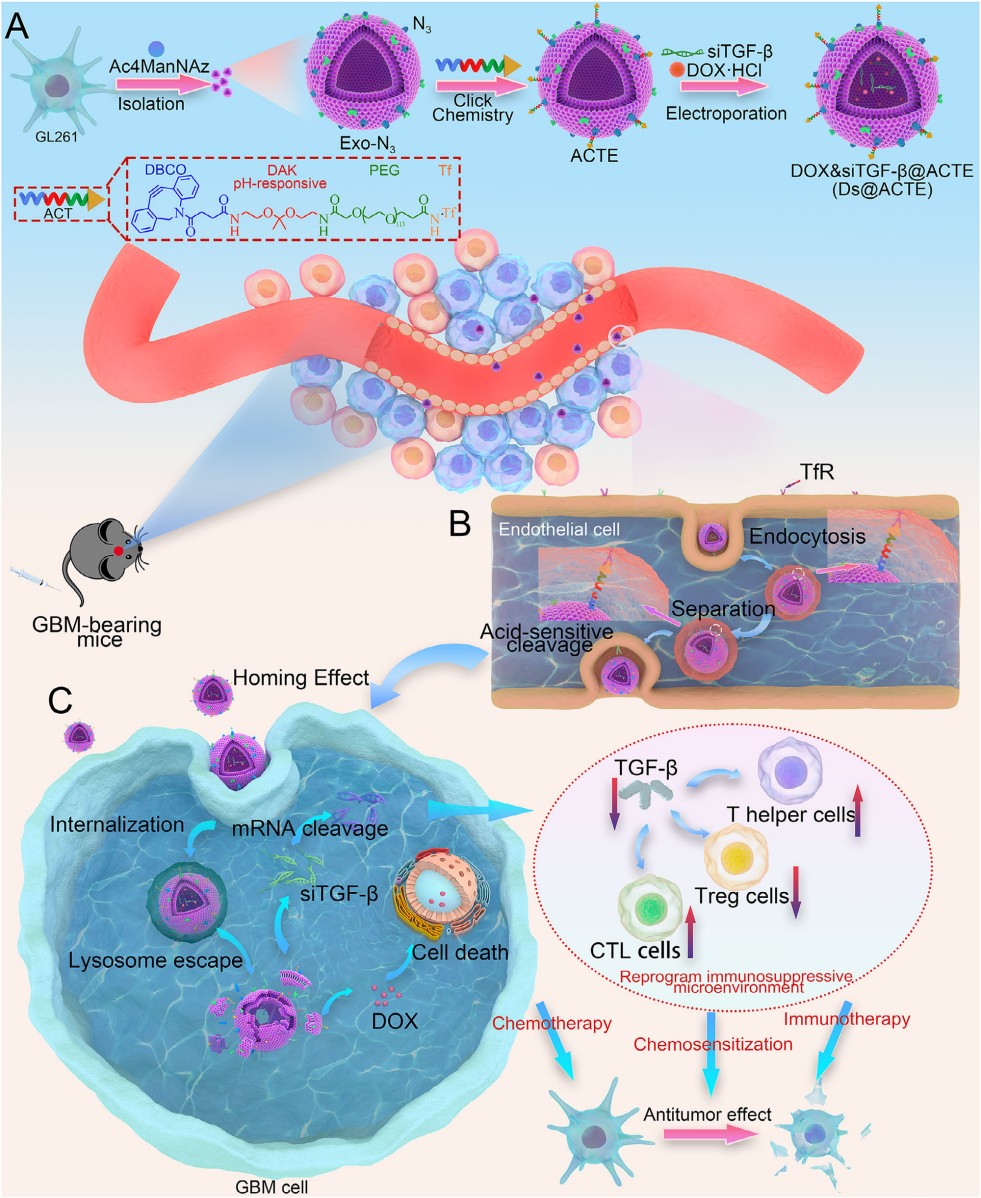
Schematic illustration of the preparation, GBM‐targeting delivery, and chemo‐immunotherapy of Ds@ACTE. (A) Diagram depicting the preparation procedures of Exo‐N_3_, ACTE, and Ds@ACTE. (B) Diagram depicting the mechanisms of acid‐responsive detachment of Tf modification from Ds@ACTE within endo/lysosomes of BCECs and the subsequent GBM‐targeting delivery via homing effect after transcytosis across BBB. (C) Schematic illustration of the mechanisms of Ds@Exo involved in reprogramming the GBM immunosuppressive microenvironment and sensitization of chemotherapeutic effect.

Herein, in this study, we proposed an acid‐cleavable Tf‐modified engineering exosome‐based brain‐targeting delivery system (ACTE) for the combination of RNAi therapy with chemotherapy against GBM. GL261 cells, a murine GBM cell line, were chosen and pre‐treated with Ac4ManNAz (an azido‐containing metabolic glycoprotein labeling reagent) to enable the bearing of N_3_ on the cell surface through metabolic glycoengineering. Subsequently, exosomes isolated from Ac4ManNAz‐treated GL261 cells also bear N_3_ on their membrane, generated as N_3_‐bearing exosome (Exo‐N_3_). Exo‐N_3_ was conjugated with dibenzocyclooctyne‐diamino ketal‐poly(ethylene glycol)‐transferrin (DBCO‐DAK‐PEG‐Tf), referring as acid‐cleavable Tf (ACT) ligand, via bioorthogonal reaction to generate ACTE. Doxorubicin (DOX) and siTGF‐β were further loaded into ACTE via electroporation, yielding the Ds@ACTE (Figure 1). After intravenous administration, Ds@ACTE could specifically recognize TfR on the luminal (blood) side of the BBB. Upon entering the lysosome of BCECs, Ds@ACTE underwent acid‐triggered detachment of Tf, resulting in the separation of Ds@Exo from the Tf‐TfR complex within the endosomes/lysosomes. The separated Ds@Exo exhibited enhanced lysosomal escape and transcytosis into the brain parenchyma. More importantly, exosomes originating from GL261 cells possessed a homing effect on GBM cells, resulting in targeted delivery of DOX and siTGF‐β. The release of DOX could exert cytotoxic effects on GBM cells and initiate anti‐GBM immune response by releasing tumor‐associated antigens. Meanwhile, siTGF‐β could reprogram the immunosuppressive microenvironment of GBM by downregulating TGF‐β expression, ultimately re‐sensitizing chemotherapeutic response and potentiating anti‐GBM immune response. In vitro studies showed an enhanced BBB transcytosis efficiency of Ds@ACTE compared to an acid‐nonresponsive delivery system. In vivo studies showed that Ds@ACTE could significantly target the GBM site with higher accumulation and efficiently reprogram the immunosuppressive microenvironment of GBM. More importantly, Ds@ACTE significantly delayed GBM growth and prolonged the survival time of orthotopic GL261‐bearing mice. The effectiveness of this strategy not only can provide thinking for designing a more functional engineered exosome‐based brain‐targeting system but also explore an effective combination therapy for GBM patients.

## 2 RESULTS AND DISCUSSION

### 2.1 Preparation and characterization of ACTE

**FIGURE 2:**
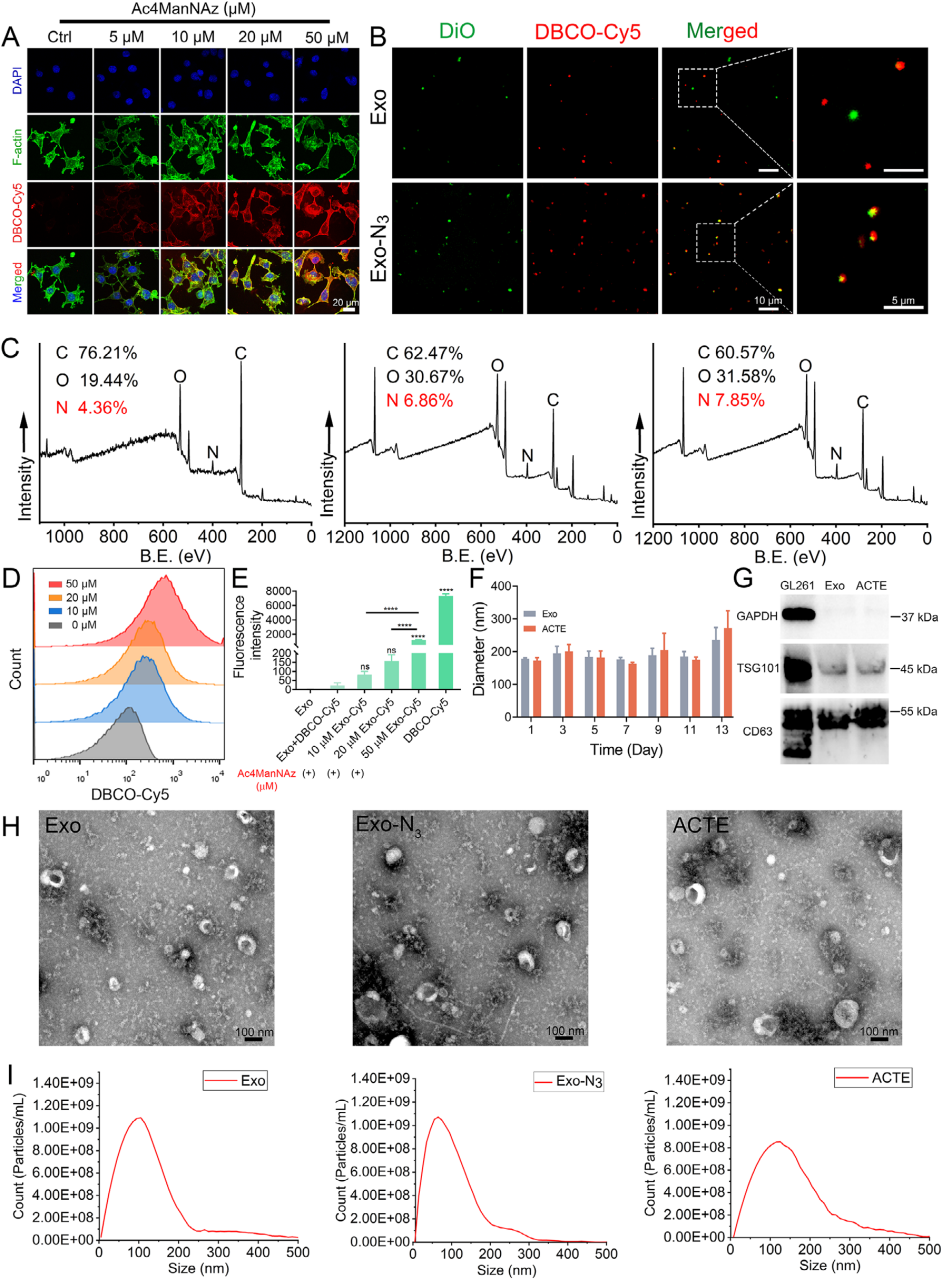
In vitro preparation and characterization of ACTE. (A) CLSM images of DBCO‐Cy5 labeled GL261 cells after pre‐treatment with Ac4ManNAz (5–50 µM) for 48 h. The nucleus was stained with 4′,6‐diamidino‐2phenylindole (DAPI). Scale bar is 20 µm. (B) CLSM images of DBCO‐Cy5 labeled Exo and Exo‐N_3_. The exosomes were pre‐stained with DiO. Scale bars are 10 µm (left) and 5 µm (right). (C) XPS survey spectrum of Exo, Exo‐N_3,_ and Exo‐DBCO respectively. (D) Nanoflow cytometry analysis of pristine Exo and Exo‐N_3_ from GL261 cells pre‐treatment with Ac4ManNAz (10–50 µM) for 48 h after incubation with DBCO‐Cy5 (10 µM). (E) Fluorescence intensity of pristine Exo and Exo‐N_3_ from GL261 cells pre‐treatment with Ac4ManNAz (10–50 µM) for 48 h, ns, not significant and ****p < 0.0001 represent statistical significance versus Exo+DBCO‐Cy5 (*n* = 3). (F) Hydrodynamic size of Exo and ACTE after storage at −20°C at different time intervals (*n* = 3). (G) Western blot analysis of CD63, TSG101 and glyceraldehyde‐3‐phosphate dehydrogenase (GAPDH) expression in GL261 cell, Exo and ACTE. (H) TEM images of Exo, Exo‐N_3_, and ACTE. Scale bar is 100 nm. (I) NTA results of Exo, Exo‐N_3_, and ACTE. All bars represent mean ± SD.

The prerequisite for utilizing biorthogonal reaction to engineer exosomes is the presence of N_3_ on the surface of the exosome. To validate the successful metabolic glycoengineering, GL261 cells were pre‐treated with Ac4ManNAz for 24, 48, and 72 h at different concentrations. Subsequently, DBCO‐Cy5, a fluorescence probe, was utilized to detect the level of surface N_3_. Confocal laser scanning microscopy (CLSM) imaging demonstrated that the fluorescence signal of Cy5 increased with the increase of Ac4ManNAz concentration, with a maximum signal at 50 µM after 48 h of incubation (Figure 2A). Meanwhile, increasing the incubation time also increased the fluorescence signal of Cy5 when incubated at the same concentration (Figure S1). Moreover, the fluorescence signal of Cy5 was in good co‐localization with the fluorescence signal of F‐actin, a typical membrane labeling fluorescence probe. The results indicated the successful metabolic glycoengineering and azidation level at the cell surface was in a concentration‐dependent and time‐dependent manner. Furthermore, the cell viability of GL261 cells showed no significant difference after incubation with Ac4ManNAz for 24, 48, and 72 h, even at the highest concentration of 50 µM, suggesting the low cytotoxicity of Ac4ManNAz (Figure S1).

Next, we assessed whether the exosomes isolated from Ac4ManNAz‐treated GL261 cells carried N_3_. Quantification by nanoparticle tracking analysis (NTA) showed no significant difference in the quantity of exosomes produced by Ac4ManNAz‐treated cells compared to untreated cells, suggesting Ac4ManNAz has no significant influence on exosome biogenesis and secretion (Figure S1). To confirm the successful carrying of N_3_ on the surface of exosomes, the exosomes isolated from both Ac4ManNAz‐treated and untreated cells were directly incubated with 10 µM DBCO‐Cy5. Fluorescence intensity determined by both nanoflow cytometry and microplate reader demonstrated that exosomes isolated from Ac4ManNAz‐treated cells exhibited higher fluorescence intensity than exosomes isolated from untreated cells, and was proportional to incubation concentration (Figure 2D,E). Additionally, 3,3′‐dioctadecyloxacarbocyanine per‐chlorate (DiO), a hydrophobic fluorescence probe, was used for labeling the lipid bilayer of exosomes. CLSM imaging of exosomes from Ac4ManNAz‐treated cells revealed an excellent co‐localization of the green fluorescence signal (DiO) with red fluorescence signal (DBCO‐Cy5). By contrast, colocalization of red and green fluorescence signals was not observed in the exosome from untreated cells (Figure 2B). Notably, exosomes extracted from GL261 cells treated with 50 µM Ac4ManNAz exhibited a higher degree of azidation. These results suggested that N_3_ was successfully packaged onto the surface of exosomes during biogenesis and the azidation level was positively correlation with Ac4ManNAz concentration. It can be assumed that the package of N_3_ on the surface of exosomes would increase the surface nitrogen (N) ratio. Therefore, we further determined the composition and proportion of elements on the surface of exosomes using surface X‐ray photoelectron spectroscopy (XPS). The results showed that the surface N proportion of exosomes from Ac4ManNAz‐treated cells increased from 4.36% to 6.86% compared to exosomes from untreated cells (Figure 2C). Moreover, we deconvoluted the detailed spectra of C1s, N1s, and O1s within the XPS full spectrum, revealing an extra N≡N peak in the N1s spectrum of Ac4ManNAz‐treated cellular exosomes, and an additional π–π peak in the C1s spectrum following the reaction with DBCO‐NHS (Figure S2). All these results served as a proof‐of‐concept to confirm the existence of N_3_ on the surface of exosomes from Ac4ManNAz‐treated cells and the N_3_ was able to take place biorthogonal reaction with DBCO.

To prepare ACTE, the ACT (DBCO‐DAK‐PEG‐Tf) was first synthesized by conjugating DBCO‐NHS with Tf via DAK, an acid‐liable linker^[^ ^33^ ^]^ (Figure S3). Proton nuclear magnetic resonance (^1^H‐NMR) and matrix‐assisted laser desorption/ionization time of flight (MALDI‐TOF) mass spectrometry verification suggested that acid‐cleavable DBCO‐DAK‐PEG‐Tf was successfully synthesized, as well as acid‐noncleavable Tf (DBCO‐PEG‐Tf, referring as ANT) (Figures S3–S6). DBCO‐DAK‐PEG‐Tf was incubated with Exo‐N_3_ to obtain ACTE via biorthogonal reaction, the proportion of both determined by flow cytometry (Figure S7). To provide comprehensive parallel controls, Exo‐N_3_ decorated with DBCO‐PEG‐Tf (ANT) and DBCO‐PEG (P) were also prepared to obtain ANTE and PE, respectively. Exosomes (Exo) without any modification were also prepared as a control. Western blot (WB) analysis indicated that the typical markers of exosomes, CD63 and tumor susceptibility gene 101 (TSG101), were displayed on ACTE and Exo (Figure 2G). Additionally, transmission electron microscopy (TEM) imaging showed that Exo, Exo‐N_3_, and ACTE were all typical cup‐shaped or donut morphology with a similar diameter of approximately 100 nm (Figure 2H). NTA results further confirmed a similar size distribution with a peak diameter of approximately 100 nm between ACTE and Exo, indicating that the decoration of ACT did not alter the size and morphology of exosomes (Figure 2I). Although the zeta potential of Exo, Exo‐N_3_, and ACTE demonstrated a slight change after azidation as well as bioorthogonal conjugation compared to untreated exosomes, the zeta potential of ACTE remains negative and approximately neutral, which is beneficial for circumventing protein absorption during circulation and thus prolonging blood circulation time (Figure S8). Meanwhile, we evaluated the stability of Exo and ACTE after storage at −20°C and ACTE stored in PBS and 10% FBS at 4°C. The data showed that the hydrodynamic size and the zeta potential revealed no notable differences within 11 days (Figure 2F, Figure S9). Additionally, we measured the drug release profiles of Exo loaded with DOX and siRNA in pH 5.0 and 7.4 PBS to simulate the acidic intracellular environment and physiological extracellular environment, respectively. The release profile of either DOX or siRNA demonstrated a pH‐dependent drug release behavior, which was beneficial for reducing non‐specific drug release in normal tissues (Figure S10). Collectively, these data confirmed that ACTE was successfully prepared without significant influence on the structural, functional integrity, as well as stability of exosomes.

### 2.2 In vitro uptake evaluation of ACTE

**FIGURE 3:**
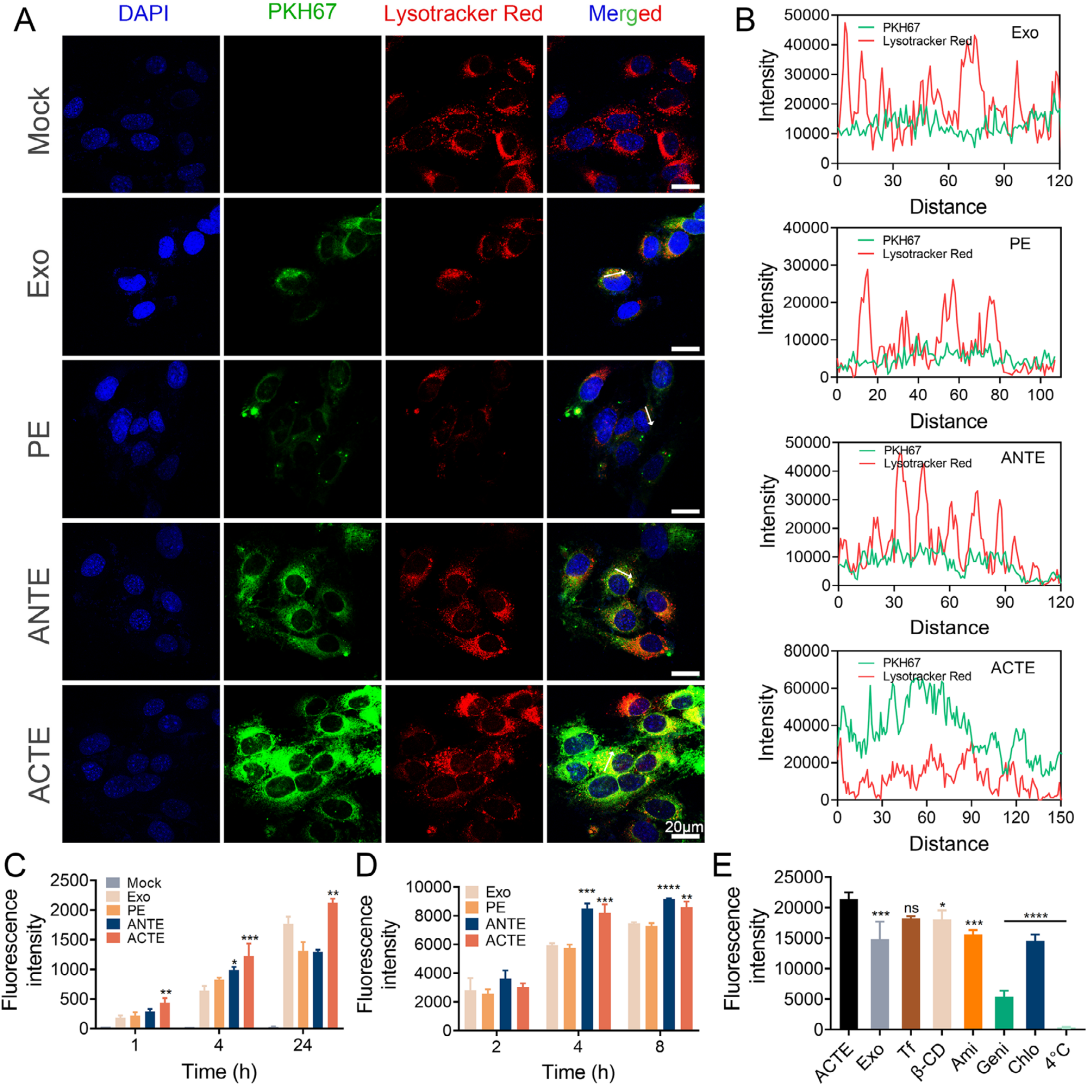
In vitro evaluation of cellular uptake. (A) Representative CLSM images of bEnd.3 cells after incubation with different engineered exosomes for 4 h. Scale bar is 20 µm. (B) Intensity curve along the indicative profile line in (A). (C) Semi‐quantitative analysis of cellular uptake of different engineered exosomes by bEnd.3 cells after incubation for 1, 4, and 24 h quantified by confocal images, *p < 0.05, **p < 0.01, and ***p < 0.001 represent statistical significance versus Exo (*n* = 3). (D) Cellular uptake of different engineered exosomes by bEnd.3 cells after incubation for 2, 4, and 8 h using flow cytometry analysis, **p < 0.01, ***p < 0.001, and ****p < 0.0001 represent statistical significances versus Exo respectively (*n* = 3). (E) Internalization mechanism exploration of ACTE by bEnd.3 cells after pre‐treatment with different inhibitors, *p < 0.05, ***p < 0.001, and ****p < 0.0001 represent statistical significance versus ACTE without any inhibitor's treatment (*n* = 3). All bars represent mean ± SD.

The internalization of ACTE by endothelial cells is the prerequisite for efficient BBB transcytosis. Thus, we next evaluated the cellular uptake of ACTE by bEnd.3 cells, a murine brain endothelial cell line, which can mimic BCECs functionality. After incubation for 1 h, CLSM images of bEnd.3 cells treated with different PKH67‐labelled exosomes revealed clear green fluorescence signals, indicating that exosomes could be internalized into bEnd.3 cells (Figure S11). Meanwhile, the fluorescence signal of exosomes showed a good co‐localization with the fluorescence signal of the lysosome, suggesting the internalization of exosomes was mainly through the endocytic pathway. Importantly, after incubation for 4 h, ACTE exhibited a much stronger fluorescence signal than PE and Exo, suggesting that Tf modification effectively facilitated cellular uptake through TfR‐mediated endocytosis (Figure 3A). To directly evaluate the cellular uptake and lysosome escape efficiency of ACTE and other control groups, we conducted a semi‐quantitative analysis of the co‐localization between exosome and lysosome fluorescence signals. Compared to Exo, PE, and ANTE, ACTE exhibited weaker overlap between the green fluorescence of exosomes and the red fluorescence of lysosomes at 4 h, suggesting ACTE was more likely to escape from lysosomes (Figure 3B). Quantitative analysis of fluorescence intensity calculated from CLSM images showed a time‐dependent cellular uptake of all exosome formulations and the cellular uptake of ACTE by bEnd.3 cells was stronger than control groups (Figure 3C and Figure S12). To directly prove whether siRNA could be successfully transfected into cells using these exosomes, we first encapsulated Cy5‐siRNA into different exosome formulations and transfected GL261 cells for 4 h. CLSM demonstrated clear fluorescence intensity in GL261 cells transfected with different formulations, with a much higher intensity of Cy5‐siRNA‐loaded ACTE compared to other formulations, suggesting that ACTE could successfully deliver siRNA into cells with higher efficiency. Meanwhile, the fluorescence intensity of the Cy5‐siRNA‐loaded ACTE in the GL261 cells after acid treatment was weaker than that before acid treatment, as well as the ANTE group, whereas comparable to the PE group and Exo group (Figure S13). This result suggested that pre‐acidification can lead to the detachment of outer Tf modification, thereby reducing the cellular uptake of ACTE by GL261 cells. Furthermore, flow cytometry analysis demonstrated a similar cellular uptake between ANTE and ACTE, both of which were significantly higher than Exo and PE (Figure 3D), further confirming that Tf modification could facilitate cellular uptake. Collectively, all these results suggested that ACTE was more likely to be internalized by bEnd.3 cells and to escape from lysosomes, which was probably owing to that the ACT modification could be detached from ACTE and thus enhance lysosomal escape compared to ANT modification.

To elucidate the underlying mechanisms involved in the cellular uptake of ACTE, we pre‐incubated bEnd.3 cells with different inhibitors, including chlorpromazine (Chlo), β‐cyclodextrin (β‐CD), amiloride (Ami), and genistein (Geni), which inhibit clathrin‐mediated endocytosis, lipid raft‐mediated endocytosis, micropinocytosis, and vesicle‐mediated endocytosis, respectively.^[^ ^34^ ^]^ Notably, after pro‐treatment with the genistein, the cellular uptake of ACTE reduced to 25.3% as compared to the ACTE without any treatment, emphasizing the predominant role of caveolin‐mediated endocytosis in ACTE internalization (Figure 3E, Figure S14). Moreover, pre‐treatment with free Tf reduced the cellular uptake to 85.3%, which can be attributed to competitive inhibition by Tf, further underscoring the involvement of TfR‐mediated endocytosis in ACTE cellular uptake. Following pre‐incubation with chlorpromazine, amiloride and β‐cyclodextrin, the cellular uptake of ACTE reduced to 68.1%, 73.1%, and 84.3%, respectively, indicating that clathrin‐mediated endocytosis, micropinocytosis, and lipid raft‐mediated endocytosis were also involved in the exosome internalization. These findings collectively indicated the involvement of multiple pathways in the internalization of ACTE by bEnd.3 cells.

### 2.3 Evaluation of in vitro BBB transcytosis

**FIGURE 4:**
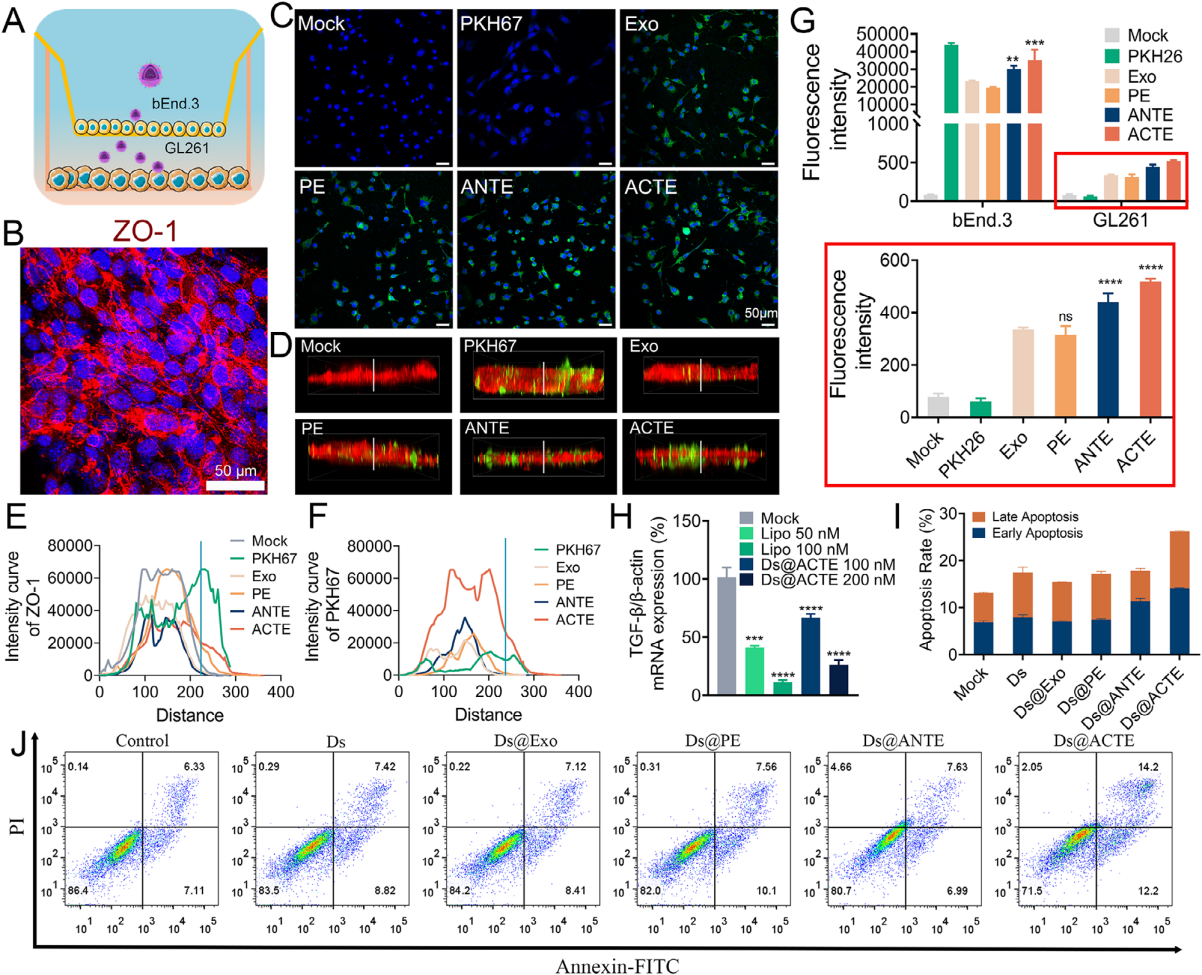
Evaluation of in vitro BBB transcytosis and pharmacological action. (A) Schematic illustration of Transwell model. (B) CLSM images of bEnd.3 monolayer after immunofluorescence staining with anti‐ZO‐1 antibody. Scale bar is 50 µm. (C) Confocal images of GL261 cells in the acceptor chamber of the Transwell model after the introduction of different engineered exosomes for 6 h. Scale bar is 50 µm. (D) 3D confocal images of bEnd.3 monolayer in the donor chamber of Transwell model after the introduction of different engineered exosomes for 6 h. (E) Intensity curve of ZO‐1 at the indicative location and distance from (D). (F) Intensity curve of PKH67 at the indicative location and distance from (D). (G) Cellular uptake of different engineered exosomes by bEnd.3 membranes in the donor chamber and by GL261 cells in the acceptor chamber after introduction into Transwell model for 6 h using flow cytometry analysis, **p < 0.01, ***p < 0.001, and ****p < 0.0001 represent statistical significances versus Exo respectively (*n* = 3). (H) Relative expression of TGF‐β mRNA in GL261 cells. Cells were treated with Ds@ACTE and siTGF‐β@Lipo2000, ***p < 0.001 and ****p < 0.0001 represent statistical significances versus Mock respectively (*n* = 3). (I) Quantitative analysis of apoptosis and necrosis of GL261 cells after staining with Annexin V‐FITC and PI (*n* = 3). (J) Representative flow cytometry analysis of GL261 cells in the acceptor chamber after staining with Annexin V‐FITC and PI, Transwell models were treated with different engineered exosomes for 24 h.

To evaluate the BBB transcytosis ability of ACTE, we established an in vitro BBB model with Transwell model, where bEnd.3 cells were seeded into the upper chamber and GL261 cells were seeded into the bottom chamber (Figure 4A). Following establishment, the bEnd.3 monolayer on the upper chamber was performed an immunofluorescence staining with ZO‐1, a typical tight junction‐associated protein, to confirm the integrity and tightness of the in vitro BBB model (Figure 4B). Subsequently, ACTE and control groups were added to the upper chamber for 6 h, CLSM images of GL261 cells in the bottom chamber demonstrated higher fluorescence signals of both ACTE and ANTE than PE and Exo (Figure 4C). This result suggested that Tf coating was beneficial for assisting the transcytosis across bEnd.3 monolayers and further assisting cellular uptake by GL261 cells. In comparison, ACTE possessed a relatively higher fluorescence signal than ANTE, indicating the involvement of ACT in promoting more transcytosis across bEnd.3 monolayers compared to ANT. Furthermore, after penetrating the bEnd.3 monolayers, ACTE could further target GL261 cells owing to its homing effect. It was worth noting that negligible green fluorescence in GL261 cells was observed after incubation with free PKH67 dye in the upper chamber, indicating that the bEnd.3 monolayer effectively prevented the penetration of small molecules. To gain a deeper understanding of the transcytosis behavior of different exosome formulations in the BBB model, we subsequently performed 3D reconstruction of both the fluorescence signal of exosomes and ZO‐1 (Figure 4D). The 3D images revealed that ACTE exhibited greater penetration depth of green fluorescence signals compared to ANTE, PE, and Exo, further confirming the excellent transcytosis ability of ACTE. The mean fluorescence intensity along the bEnd.3 monolayers from top to bottom were quantitatively analyzed. The intensity curve of PKH67 of ACTE indicated an obviously longer extension than that of other engineered exosomes but the intensity curve of ZO‐1 showed no obvious difference (Figure 4E,F). Moreover, after incubation for 6 h, flow cytometry analysis demonstrated that the cellular uptake of Tf‐modified engineered exosomes by bEnd.3 monolayer in the upper chamber was higher than unmodified exosomes, while still lower than free PKH26. Despite of highest cellular uptake of free PKH26 in bEnd.3 monolayer, the uptake by GL261 cells in the bottom chamber was lowest. Similarly, the cellular uptake of ACTE by GL261 cells was higher than ANTE, PE, and Exo (Figure 4G). These results collectively indicated that the exceptional in vitro BBB transcytosis ability of ACTE might serve as a promising candidate for efficient BBB transcytosis in vivo.

Next, we utilized electroporation to load DOX and siTGF‐β into ACTE, generating Ds@ACTE with both gene silencing and cell‐killing capabilities. We investigated the gene‐silencing activity of Ds@ACTE in GL261 cells. After incubation for 24 h, the real‐time quantitative PCR (RT‐qPCR) analysis showed that treatment with Ds@ACTE (at a siRNA concentration of 100 nM) achieved approximately 35% knockdown of TGF‐β1 mRNA. Meanwhile, when increasing the siRNA concentration to 200 nM, Ds@ACTE exhibited a gene silencing efficiency of 75%, comparable to the control group (siTGF‐β@Lipo2000) (Figure 4H). Moreover, to mimic the apoptosis‐inducing ability of Ds@ACTE and other control groups on GL261 cells after transcytosis through BBB, the in vitro Transwell model was used and GL261 cells in the bottom chamber was performed a Annexin‐FITC/PI double staining. After incubation for 24 h, the early and late apoptosis percentage of GL261 cells in bottom chamber in Ds@ACTE group were 12.2% and 14.2%, respectively, surpassing DOX&siTGF‐β@ANTE (Ds@ANTE) (6.99%, 7.63%), DOX&siTGF‐β@PE (Ds@PE) (10.1%, 7.56%), Ds@Exo (8.41%, 7.12%), and free DOX&siTGF‐β (Ds) (8.82%, 7.42%) (Figure 4J). Quantitative analysis of total apoptosis percentages revealed the highest percentage of 26.2 ± 0.28% after treatment with Ds@ACTE, which was higher than that treatment with Ds@ANTE (17.8 ± 1.15%), Ds@PE (17.2 ± 0.64%), and Ds@Exo (15.4 ± 0.16%) (Figure 4I). These results collectively indicated that Ds@ACTE could induce a higher apoptosis rate in GL261 cells, confirming its enhanced transcytosis capability. These results demonstrated that Ds@ACTE possessed excellent BBB permeability as well as enhanced gene silencing and chemotherapeutic killing abilities.

### 2.4 In vivo distribution of ACTE

**FIGURE 5:**
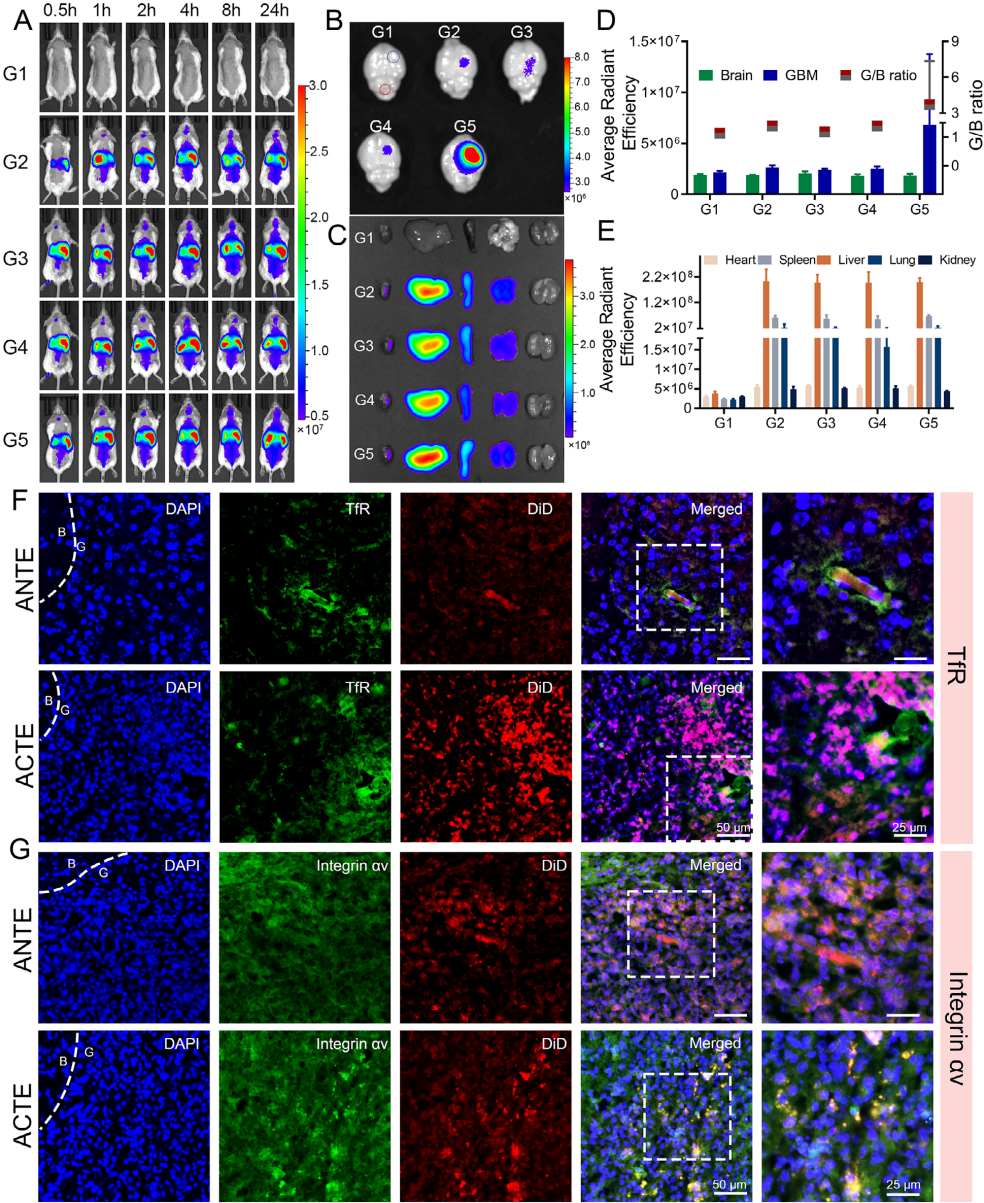
Evaluation of in vivo distribution and GBM‐targeting efficiency of ACTE. (A) Living images of GL261‐bearing mice after intravenous injection with ACTE and control‐engineered exosomes at different time intervals. G1 to G5 represents PBS, Exo, PE, ANTE and ACTE, bar represents radiant efficiency from 5.0 × 10^6^ to 3.0 × 10^7^ [p s^−1^ cm^−2^ sr^−1^]/[µW cm^−2^]. (B) Ex vivo imaging of brains collected at 24 h after injection, blue circle represents glioma site, red circle represents normal brain parenchyma, bar represents radiant efficiency from 2.7 × 10^6^ to 8.0 × 10^6^ [p s^−1^ cm^−2^ sr^−1^]/[µW cm^−2^]. (C) Ex vivo imaging of major organs collected at 24 h after injection, bar represents radiant efficiency from 1.0 × 10^7^ to 3.0 × 10^8^ [p s^−1^ cm^−2^ sr^−1^]/[µW cm^−2^]. (D) The left column represents the semi‐quantitative date of the fluorescence signal at the indicative site from (B), right column represents G/B ratio of different exosomes (*n* = 3). (E) Semi‐quantitative data of the fluorescence signal of major organs from (C) (*n* = 3). (F) Fluorescence distribution of ANTE and ACTE at glioma site after intravenous administration for 24 h, GBM slices were immune‐stained with anti‐TfR antibody. Scale bar is 50 µm (left) and 25 µm (right). (G) Fluorescence distribution of ANTE and ACTE at glioma site after intravenous administration for 24 h, GBM slices were immune‐stained with anti‐integrin αv antibody. Scale bar is 50 µm (left) and 25 µm (right).

To investigate the GBM‐targeting specificity and accumulation capability of engineered exosomes, we established an orthotopic GBM‐bearing mice model using GL261 cells. 14 days after cell implantation, 1,1′‐Dioctadecyl‐3,3,3′,3′‐Tetramethylindodicarbocyanine (DiD)‐labeled engineered exosomes (Exo, PE, ANTE, and ACTE) were intravenously injected. Subsequently, the whole‐body fluorescence signal was recorded using an in vivo living imaging system over a 24 h period. At 1 h post‐injection, the fluorescence signal of ACTE, ANTE, and PE was observed at the brain site (Figure 5A). Moreover, the fluorescence signal of all DiD‐labeled exosome formulations gradually increased over time. By comparison, at 24 h post‐injection, the signal of ACTE was slightly higher than that of ANTE, PE, and Exo, underscoring ACT modification could specifically recognize TfR on BBB endothelium and thus effectively mediated brain‐targeted delivery. To gain a direct verification of the GBM‐targeting ability of ACTE, all organs were harvested at 24 h for ex vivo imaging. Ex vivo imaging of brains showed a much stronger fluorescent signal of ACTE pinpointing the GBM site than control groups, which was supported by the quantitative analysis (Figure 5B,D). Additionally, we introduced a G/B ratio to assess the targeting specificity of ACTE, calculated as the ratio of fluorescence signal in the GBM region to that in normal brain regions. As expected, ACTE exhibited a G/B ratio of 1.64 ± 0.47, which was significantly higher than Exo (1.38 ± 0.14), PE (1.18 ± 0.07), and ANTE (1.38 ± 0.21) (Figure 5D). These data collectively demonstrated that ACTE possesses higher GBM‐targeting selectivity compared to control groups. Meanwhile, the decoration of ACT modification was beneficial for improving accumulation at the GBM site compared to ANT modification, mainly owing to that the ACT could facilitate lysosome escape and thus reduce BBB entrapment. Furthermore, ex vivo imaging of normal organs and semi‐quantitative analysis indicated that these engineered exosomes were primarily distributed in the liver, suggesting the liver was the major organ involved in their clearance (Figure 5C,E). To determine if ACTE effectively targets glioma sites, we conducted immunofluorescence staining of brain slices with anti‐TfR and anti‐integrin αv antibodies. The immunofluorescence staining of TfR revealed a poor co‐localization of fluorescence signals between ACTE and TfR at the glioma site, whereas a better co‐localization of fluorescence signals between ANTE and TfR (Figure 5F). By contrast, immunofluorescence staining of integrin αv exhibited a better co‐localization of fluorescence signal between ACTE with integrin αv at the glioma site compared to that between ANTE with integrin αv (Figure 5G). These findings strongly validated our hypothesis that acid‐responsive ACTE could lose its ACT modification during the transcytosis across BBB endothelium, allowing the separated exosomes to further home to GBM cells probably via recognizing integrin αv overexpressed on GBM cells. In contrast, the acid‐nonresponsive ANTE was unable to lose ANT modification, which still played a dominant role in mediating GBM‐targeting delivery, leaving the homing effect a less important role. Moreover, the fluorescence signals of Exo and PE demonstrated a good colocalization with integrin αv, further confirming the excellent homing effect of GL261‐derived exosomes (Figures S15,S16). More importantly, the fluorescence signal of ACTE was significantly stronger than those of control groups, suggesting ACTE could deliver to the GBM site in an increased amount, which was mainly due to the synergistic effect of ACT in promoting transcytosis through the BBB as well as the intrinsic homing effect of GL261‐derived exosomes.

### 2.5 In vivo anti‐glioma effect of ACTE

**FIGURE 6:**
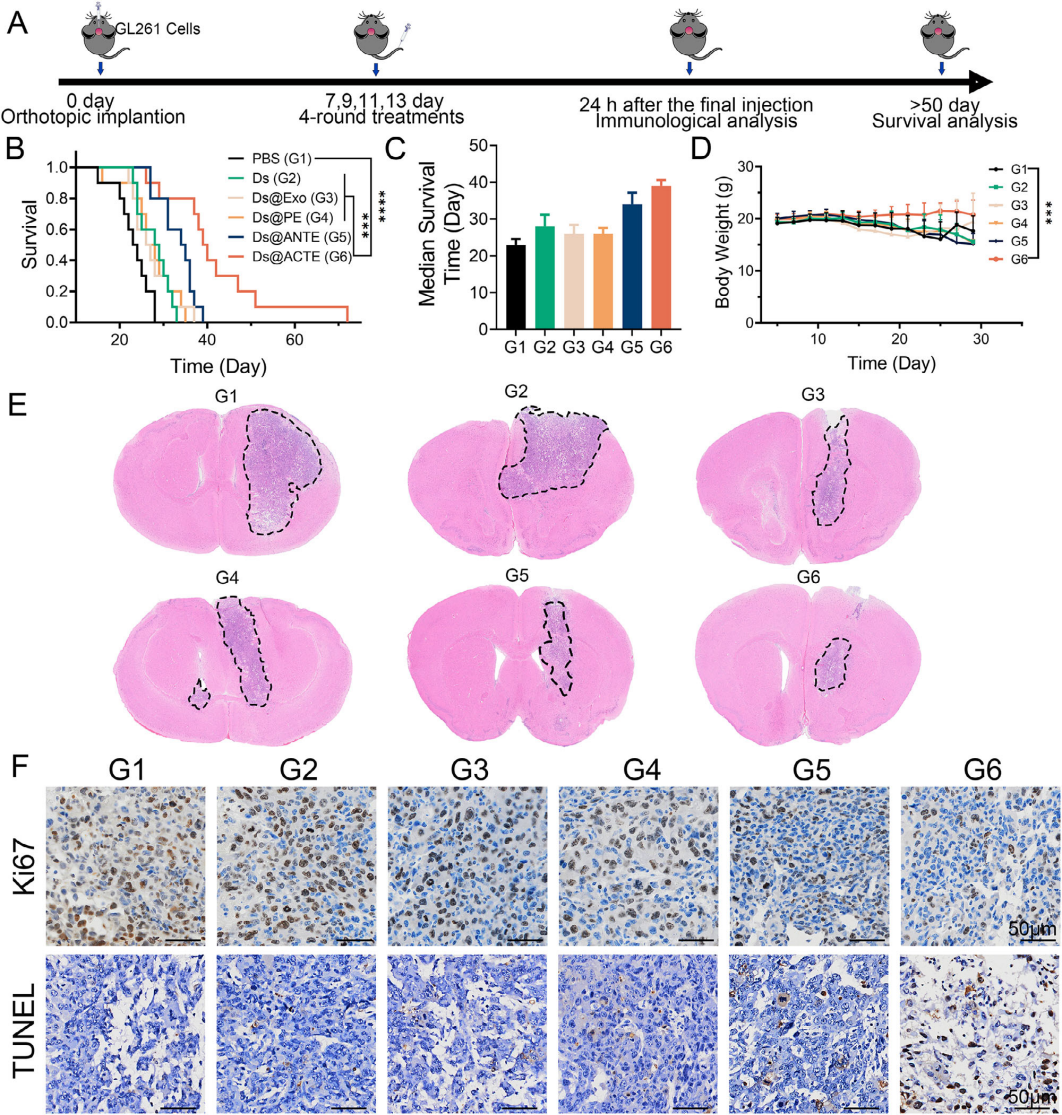
In vivo anti‐GBM efficacy of Ds@ACTE. (A) Schematic diagram of the treatment schedule and experimental design. (B) Survival curve of GL261 GBM‐bearing mice after intravenous administration of different exosomes using Kaplan–Meier analysis (*n* = 10). ***p < 0.001, ****p < 0.0001. (C) Median survival time of GL261 GBM‐bearing mice after intravenous administration of different exosomes. Error bars represent the mean ± SEM. (D) Body weight recording within 30 days after implantation (*n* = 10). ***p < 0.001. (E) H&E staining of the GL261 GBM‐bearing brains collected on the day after the last treatment, the dotted regions represent GL261 cells. (F) Ki67 staining and TUNEL staining of the GL261 GBM‐bearing brains collected on the day after the last treatment. Scale bar is 50 µm.

Encouraged by both in vitro and in vivo performance, we proceeded to investigate the in vivo anti‐GBM effect of Ds@ACTE by monitoring the survival duration of the GL261‐bearing mice (Figure 6A). Compared to the control treatment, treatment with Ds@ACTE significantly prolonged the survival duration of mice (Figure 6B). It should be noted that treatment with Ds@ANTE only received modest survival benefits compared to Ds@PE, Ds@Exo, and free DOX&siTGF‐β. Specifically, the median survival time further supported this finding that GL261‐bearing mice treated with Ds@ACTE exhibited a longest median survival time of 39 days, which was significantly longer than Ds@ANTE group (34 days), Ds@PE group (26 days), Ds@Exo group (26 days), free DOX&siTGF‐β group (28 days), and PBS group (23 days) (Figure 6C and Table S1). By continuously monitoring the body weight of the mice, a critical indicator of the therapeutic effect, it was found that treatment with Ds@ACTE led to a relatively stable body weight. However, the body weight of the mice in control groups continued to decline until their eventual death (Figure 6D and Figure S17). These data collectively underscored the potent anti‐GBM effect of Ds@ACTE compared to Ds@ANTE and other treatments.

To elucidate the underlying mechanisms behind the prolonged survival, we isolated the GL261‐bearing brains after administration and prepared paraffin sections for hematoxylin and eosin (H&E) staining (Figure 6E). In PBS and DOX&siTGF‐β groups, H&E staining demonstrated a larger area of GBM cells and denser cell nuclei, suggesting free treatment with free DOX&siTGF‐β was unable to suppress GBM growth. In contrast, treatment with Ds@Exo and Ds@PE reduced GBM cell density due to the relatively improved delivery efficiency. The density and area of GBM cells in Ds@ANTE were further reduced due to the introduction of Tf decoration could improve drug delivery efficiency. Importantly, the density was lowest in the Ds@ACTE groups compared to the control groups, further confirming its enhanced anti‐GBM efficacy. Nonetheless, H&E staining failed to provide an accurate reflection of Ds@ACTE in inhibiting GBM cell proliferation. Therefore, we employed Ki67 staining to assess GBM proliferation and TUNEL staining to evaluate apoptosis in GBM. Treatment with Ds@ACTE resulted in a notable reduction in proliferation of GBM cells and an increase in apoptosis of GBM cells compared to the control group (Figure 6F). All these results suggested that Ds@ACTE could effectively suppress GBM cell proliferation and thus delay their progression, leading to prolonged survival duration compared to acid‐nonresponsive Ds@ANTE and control treatments.

Moreover, H&E staining of major organs after receiving different exosome‐based treatments showed no significant pathological damage (Figure S18). Unlike treatment with free DOX which might lead to cardiac toxicity, treatment with exosome‐based formulations showed negligible cardiac toxicity, underscoring the potential of exosome‐based formulations could reduce the distribution of DOX in the heart and also release DOX slowly. Additionally, measurements of five serum biochemical markers, including alanine transaminase (ALT), aspartate transaminase (AST), lactate dehydrogenase (LDH), urea (UA), and triglycerides (TRIG), showed no significant differences among the treatment groups (Figure S19). Collectively, these results indicated the good biocompatibility of Ds@ACTE and control exosome‐based formulations.

### 2.6 Enhanced anti‐GBM immune response of Ds@ACTE

**FIGURE 7:**
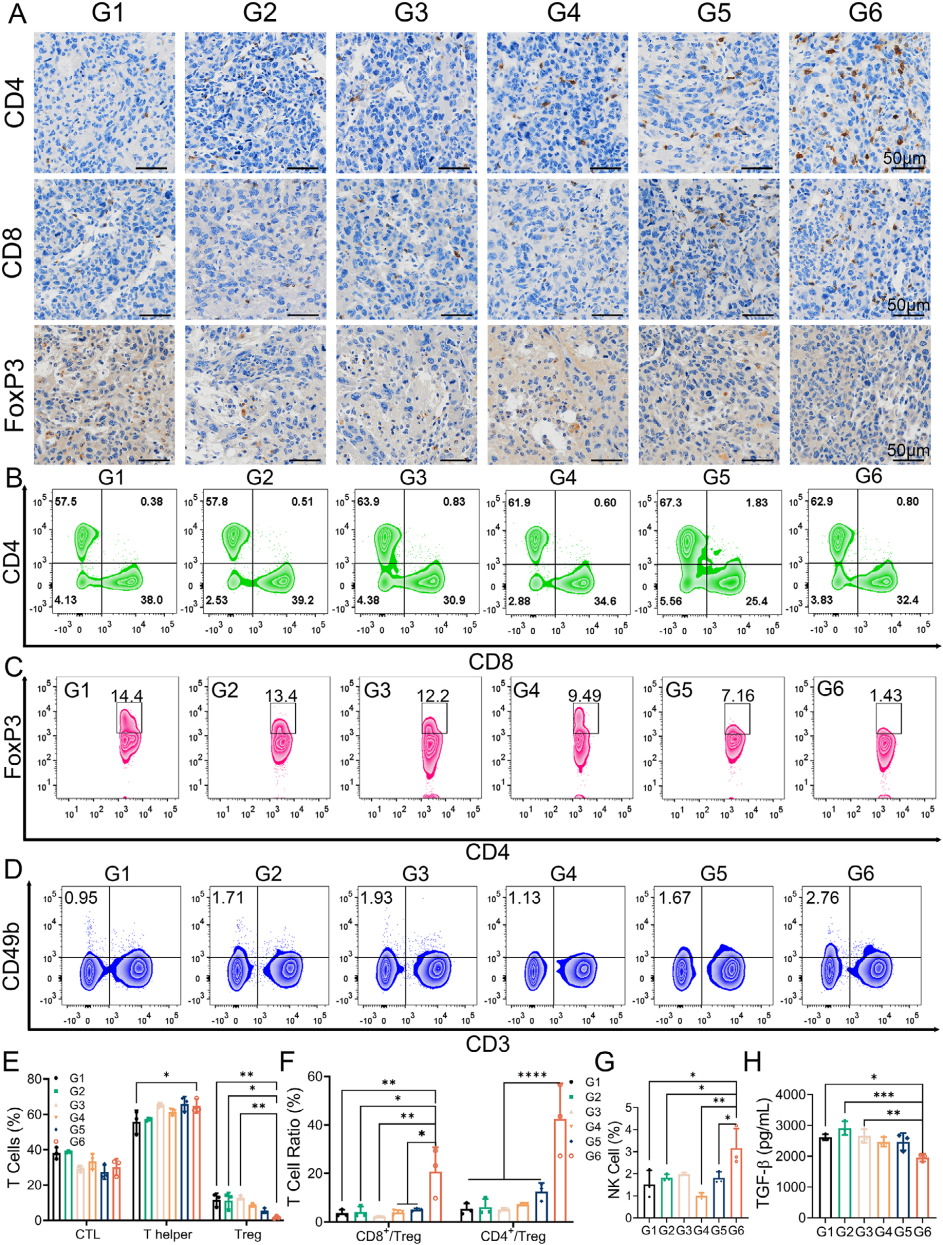
Enhanced anti‐glioma immune effect of Ds@ACTE. (A) Expression of CD4^+^ T cells, CD8^+^ T cells, and FoxP3^+^ Treg cells in GBM sections receiving different treatments. Scale bar is 50 µm. G1 to G6 represents PBS, Ds, Ds@Exo, Ds@PE, Ds@ANTE, and Ds@ACTE, respectively. (B) Representative flow cytometry analysis of CTL cells (gated on CD3^+^CD4^−^CD8^+^ cells) and T helper cells (gated on CD3^+^CD4^+^CD8^−^ cells) in the spleen from different groups. (C) Representative flow cytometry analysis of Treg cells (gated on CD4^+^FoxP3^+^) in spleen from different groups. (D) Representative flow cytometry analysis of NK cells (gated on CD3^−^CD49b^+^) in DLNs from different groups. (E) Proportions of CD8^+^ CTL cells, CD4^+^ T helper cells, CD4^+^FoxP3^+^ Treg cells according to data in (B) and (C), *p < 0.05 and **p < 0.01 represent statistical significances versus G6 respectively (*n* = 3). (F) CD8^+^ CTL: Treg ratios and CD4^+^ helper cells: Treg ratios in the spleen upon various treatments, *p < 0.05, **p < 0.01, and ****p < 0.0001 represent statistical significances versus G6 respectively (*n* = 3). (G) Proportions of NK cells according to data in (D), *p < 0.05 and **p < 0.01 represent statistical significances versus G6 respectively (*n* = 3). (H) The regulation of TGF‐β secretion after treatment with different exosomes in vivo, *p < 0.05, **p < 0.01, and ***p < 0.001 represent statistical significances versus G6 respectively (*n* = 3).

As previously reported, TGF‐β is an immunosuppressive cytokine that can significantly dampen anti‐tumor immune response.^[^ ^35^ ^]^ Therefore, we determined whether the treatment with Ds@ACTE could downregulate TGF‐β expression and thus reverse TGF‐β‐induced immunosuppression. To elucidate the immune responses in the GBM microenvironment after exosome treatment, we prepared paraffin sections of mice glioma for immunohistochemistry (IHC) staining of CD8^+^ T cells, CD4^+^ T cells, and FoxP3^+^ Treg cells. IHC staining showed that, compared to treatment with Ds, DOX@siTGF‐β@Exo, Ds@PE, Ds@ANTE, and PBS, the proportion of CD4^+^ T cells and CD8^+^ T cells in the GBM microenvironment significantly increased in mice treated with Ds@ACTE, while the proportion of FoxP3^+^ Treg cells notably decreased (Figure 7A). These dates indicated that treatment with Ds@ACTE could significantly enhance the infiltration of CD8^+^ cytotoxic T lymphocytes (CTL) and CD4^+^ helper T cells while reducing the differentiation of Treg cells. Additionally, the diminished differentiation of Treg cells significantly disrupts their immunosuppressive function, leading to a profound reprogramming of the immunosuppressive microenvironment.

Meanwhile, on the first day after treatment, the activation of CTL (CD3^+^CD4^−^CD8^+^) and T helper cells (CD3^+^CD4^+^CD8^−^) as well as the differentiation of Treg cells (CD3^+^CD4^+^FoxP3^+^) in spleens were determined using flow cytometry (Figure 7B,C and Figure S20). Treatment with Ds@ACTE significantly reduced the Treg population (1.69 ± 0.75%), which was much lower than treatment with PBS (11.6 ± 3.77%), DOX&siTGF‐β (11.2 ± 4.65%), Ds@Exo (12.9 ± 1.24%), Ds@PE (8.54 ± 0.85%), and Ds@ANTE (5.52 ± 1.43%) (Figure 7E). In addition, treatment with Ds@ACTE exhibited a significant increase in the ratio of CD8^+^ T cells to FoxP3^+^ Treg cells and in the ratio of CD4^+^ T cells to FoxP3^+^ Treg cells compared to control treatments (Figure 7F). All these results collectively indicated that Ds@ACTE was able to significantly suppress the differentiation of Tregs, despite of modest ability to induce activation of CD8^+^ T cells and CD4^+^ T cells. The reduced differentiation of Tregs was beneficial for reprogramming the immunosuppressive microenvironment of GBM, which could reinforce the anti‐GBM immune response as exhibited with much increased CD8^+^ T cells/Tregs ratio and the CD4^+^ T cells/Tregs ratio. The enhanced immunoregulatory ability of Ds@ACTE was mainly attributed to that the ACT modification could facilitate transcytosis through BBB endothelium and thus enhance the accumulation of siTGF‐β at the GBM site. Moreover, the reprogramming of immunosuppressive GBM microenvironment re‐sensitized GBM cells to chemotherapy, as confirmed by enhanced GBM suppressing efficiency described above. Furthermore, previous studies have indicated that overexpression of TGF‐β can inhibit the activation and cytotoxicity of NK cells.^[^ ^36^ ^]^ Hence, we also assessed the proliferation of NK cells (CD3^−^CD49b^+^) in the draining lymph nodes (DLNs) (Figure 7D). It was observed that treatment with Ds@ACTE led to an increase in the NK cell population, which was higher than that in control groups (Figure 7G). Therefore, Ds@ACTE also exhibited excellent potential in activating the innate anti‐tumor immune response of NK cells to combat GBM cells. We also measured the levels of TGF‐β in the serum, and as anticipated, mice treated with Ds@ACTE exhibited a significant reduction in TGF‐β secretion (about 25% reduction compared to the untreated group) when compared to other groups (Figure 7H). These results suggested that Ds@ACTE could effectively inhibit TGF‐β expression and decrease the expression and differentiation of Treg cells in the GBM microenvironment. At the same time, Ds@ACTE could improve anti‐tumor immunity and re‐sensitize chemotherapy response by reprogramming the immune suppression microenvironment.

## 3 CONCLUSION

In summary, we successfully established an intelligent GBM‐targeting delivery system based on acid‐responsive engineered exosomes (ACTE) for the combination of RNAi therapy and chemotherapy to combat GBM. The generated ACTE was validated by an efficient BBB transcytosis and improved brain entering amounts due to the introduction of acid‐cleavable Tf (ACT) modification compared to acid‐nonresponsive ANTE both in vitro and in vivo. Moreover, ACTE also demonstrated a much better in vivo GBM‐targeting specificity owing to the intrinsic homing effect of GBM cell‐derived exosomes. After co‐loading with DOX and siTGF‐β via electroporation, the generated Ds@ACTE efficiently reprogrammed the immunosuppressive microenvironment of GBM by downregulating anti‐inflammatory TGF‐β expression, which thus re‐sensitized the chemotherapeutic effect of DOX as well as boosted DOX‐initiated immune response, resulting in a synergistic therapeutic effect. Importantly, anti‐GBM studies confirmed that Ds@ACTE significantly suppressed the GL261 cell proliferation and thus prolonged the survival time of GL261‐bearing mice compared to control treatments, without causing significant systemic cytotoxicity. The excellent GBM‐targeting delivery performance and treatment outcomes of this acid‐responsive engineered exosome‐brain targeting system provide insight into the design of more functional exosome‐brain delivery systems and hold promise for the treatment of GBM and other CNS diseases.

## 4 MATERIALS AND METHODS

### 4.1 Materials, cell lines, and animals

Doxorubicin hydrochloride (DOX·HCI) was purchased from Merck (Shanghai, China). DiO and DiD were supplied by Beyotime (Shanghai, China). PKH26 (D0030) and PKH67 (D0031) were obtained from Solarbio (Beijing, China). siTGF‐β (sense strand, 5′‐GCAACAACGCCAUCUAUGATT‐3′, antisense strand, 5′‐UCAUAGAUGGCGUUGUUGCTT‐3′) was purchased from Suzhou Ribo Life Science (Jiangsu, China). LysoTracker Red (DND‐99) and Lipofectamine 2000 (Lipo 2000) were purchased from Thermo Fisher Scientific (Waltham, USA). Anti‐TSG101 antibody (Cat. No. 67381‐1‐Ig), anti‐ZO‐1 antibody (Cat. No. 21773‐1‐AP), anti‐CD63 antibody (Cat. No. 67605‐1‐Ig), and anti‐β‐actin antibody (Cat. No. 20536‐1‐AP) were purchased from Proteintech (Chicago, USA). Anti‐TfR antibody (Cat. No. sc‐65882) was purchased from Santa Cruz Biotechnology (CA). Anti‐integrin alpha v (Cat. No. ab179475) and phalloidin‐iFluor 488 (Cat. No. ab176753) were obtained from Abcam Ltd. (Cambridge, UK). Anti‐mouse APC‐CD3 antibody (Cat. No. 17‐0031‐82), Anti‐mouse FITC‐CD4 antibody (Cat. No. 11‐0041‐82), Anti‐mouse CD49b antibody (Cat. No. 48‐5971‐82), and Anti‐mouse PE‐FoxP3 antibody (Cat. No. 12‐5773‐80) were purchased from Invitrogen (Thermo Fisher, USA). Anti‐mouse Pacific Blue‐CD8a (Cat. No. 100725) was obtained from BioLegend (California, USA). Holo‐Transferrin human and 4′,6‐diamidino‐2‐phenylindole (DAPI) were purchased from Sigma‐Aldrich (Merck, USA). Dibenzocyclooctyne‐*N*‐hydroxysuccinimidyl ester (DBCO‐NHS), cyanine5 dibenzocyclooctyne (DBCO‐Cy5), and Ac4ManNAz were purchased from Click Chemistry Tools (Arizona, USA). Amino‐poly(ethylene glycol)‐carboxyl (NH2‐PEG‐COOH, *M_W_* = 2000) and carboxyl‐poly(ethylene glycol)‐carboxyl (COOH‐PEG‐COOH, *M_W_* = 5000) were purchased from Tansh‐Tech (Guangzhou, China). *N*‐(3‐(Dimethylamino)propyl)‐*N′*‐ethylcarbodiimide hydrochloride (EDC) and *N*‐hydroxy‐succinimide (NHS) were purchased from Sigma (Saint Louis, MO, USA).

GL261 and bEnd.3 were obtained from the Chinese Academy of Science Cells Bank (Shanghai, China). All cells were cultured in DMEM with 10% v/v FBS and 1% v/v penicillin/streptomycin solution, at 37°C in a humidified atmosphere of 5% CO_2_. All experiments were performed in the logarithmic phase of cell growth.

Male C57BL/6J mice (6–8 weeks) and male BALB/c mice (6–8 weeks) were purchased from Vital River Laboratories (Beijing, China) and kept in the Beijing Institute of Technology Laboratory Animal Center, which was a specific pathogen‐free experimental animal facility. All procedures were approved by the Institutional Animal Care and Use Committee (IACUC) of the Beijing Institute of Technology and performed under the guidelines and policies.

### 4.2 Cytotoxicity assessment of Ac4ManNAz on GL261 cells

The cytotoxicity of different concentrations of Ac4ManNAz on GL261 cells at various time points was determined. GL261 cells were seeded in 96‐well plates at 5000 per well and cultured for 24 h. Subsequently, cells were incubated with different concentrations of Ac4ManNAz (0, 2, 5, 10, 20, 50, and 100 µM) for 24, 48, and 72 h. Post‐incubation, MTT solution (5 mg mL^−1^) was added to each well at a final concentration of 0.5 mg mL^−1^ and incubated with GL261 cells for another 4 h. Following this, 100 µL of DMSO solution was added to each well to dissolve the formazan crystals. The absorbance at 490 nm wavelength was measured using a SpectraMax M3 microplate reader (Molecular Devices, USA).

### 4.3 In vitro validation of azidation on GL261 cells

GL261 cells were seeded in 6‐well plates at 1 × 10^6^ cells per well and cultured for 24 h. Subsequently, the culture medium was replaced with different concentrations of Ac4ManNAz (0, 5, 10, 20, 50 µM) and incubated for 24, 48, and 72 h, respectively. Afterward, cells were washed twice with PBS and incubated in DMEM containing 10 µM DBCO‐Cy5 for 1 h. The cells were then washed twice with PBS, fixed with 4% paraformaldehyde, stained with DAPI (5 µg mL^−1^) for nuclear staining and phalloidin‐iFluor 488 for actin filaments staining, and finally mounted on slides for observation under confocal laser scanning microscopy (CLSM).

### 4.4 Exosome isolation and purification

GL261 cells were seeded in 150 mm culture dishes at 5 × 10^6^ cells per dish and allowed growth for 6 h, and then incubated with different concentrations of Ac4ManNAz for another 48 h, GL261 cells without any treatments were served as control. Subsequently, the culture medium was discarded and replaced with a fresh DMEM medium containing exosomes‐depleted FBS for an additional 48 h. Post‐incubation, the cell supernatant from different conditioned GL261 cells was harvested for exosome isolation using a previously reported ultracentrifugation method.^[^ ^37^ ^]^ In brief, the cell culture supernatant was subjected to centrifugation at 200 × *g* for 10 min, followed by centrifugation at 2000 × *g* for 10 min to remove cell debris. The obtained cell‐free supernatant was further centrifuged at 10,000 × *g* for 30 min to remove microvesicles. The supernatant was then sterile‐filtered through a 0.22 µm filter, and the filtrate was collected using a pipette. Subsequently, exosomes were pelleted by ultracentrifugation at 100,000 × *g* for 70 min at 4°C using an ultracentrifuge (Optima XPN, Beckman Coulter, USA), washed with PBS, and subjected to additional ultracentrifugation at 100,000 × *g* for 70 min to remove contaminating proteins. The isolated exosomes were resuspended in ice‐cold PBS for further study and stored at −80°C.

### 4.5 Transmission electron microscopy (TEM) analysis

Briefly, exosomes were mixed with an equal volume of 4% paraformaldehyde (PFA) for 10 min. Then, 5 µL of exosome mixture was applied onto an ultrathin copper grid with a formvar carbon film and was quiescent for 20 min. The grid (formvar face down) was washed with PBS droplets. Subsequently, the grid was placed on 1% glutaraldehyde droplets for 5 min and washed three times with PBS. Under pH 7 condition, the grid was negatively stained with 2% uranyl oxalate for 5 min, and dried at room temperature before observation. Finally, electron microscopy images were captured using TEM (Tecnai G2 F20 U‐TWIN, FEI, USA) at 80 kV.

### 4.6 Nanoparticle tracking analysis (NTA)

The size and particle number of isolated exosomes were analyzed by NTA using the ZetaView (Particle Metrix, German). Briefly, 25 µL of the pellet suspension was diluted 400‐fold with PBS and 1 mL of solution was introduced into the system. This system employs a focused laser beam on the suspension of the particles of interest. The Brownian motion of each particle was tracked across multiple frames, enabling the determination of particle size by applying the Stokes–Einstein equation. Software settings for capture and analysis were shown as follows: minimal bright = 30, minimal size = 10, maximal size = 1000, sensitivity = 65, shutter = 100.

### 4.7 Validation of azido‐carried exosomes (Exo‐N_3_)

Exo‐N_3_ was confirmed through three methodologies: CLSM, flow cytometry, and X‐ray photoelectron spectroscopy (XPS). For CLSM observation, exosomes were first labeled with 5 µM DiO for 30 min at room temperature, followed by the incubation of 10 µM DBCO‐Cy5 for another 1 h. Subsequently, the mixture was washed with PBS twice to remove residual DiO and DBCO‐Cy5. Exosomes were then purified via ultracentrifugation at 100,000 × *g* for 70 min. Finally, the co‐localization of green (DiO) and red (DBCO‐Cy5) fluorescence was visualized using CLSM (Nikon, Japanese). For flow cytometry analysis, exosomes were only incubated with 10 µM DBCO‐Cy5 for 1 h. Subsequently, the mixture was washed with PBS twice to remove residual DBCO‐Cy5. Exosomes were then purified via ultracentrifugation at 100,000 × *g* for 70 min. The fluorescence signal of DBCO‐Cy5‐labeled exosomes was quantitatively analyzed using flow cytometry (CytoFLEX S, USA). For XPS analysis, Exo, Exo‐N_3_, and Exo‐DBCO were dried by dripping them onto tin‐platinum paper at identical concentrations. These samples were subsequently used for testing directly on the machine. XPS experiments were performed on a PHI QUANTERA‐II SXM (ULVAC‐PHI, Japan) with AI Kα (*hν = *1486.6 eV). The source power and high voltage was set at 150 W and 15 kV, and pass energies of 40 eV for survey scans was used. The analysis spot size was 300 × 700 µm^2^. The data was analyzed by Multipak software with C1s = 284.6 eV as a benchmark for the binding energy correction.

### 4.8 Preparation of acid‐cleavable Tf (DBCO‐DAK‐PEG‐Tf or so‐called ACT): Synthesis of acid‐sensitive DAK [2,2′‐(propane‐2,2‐diylbis(oxy))‐diethanamine cross linker]

The acid‐labile cross‐linker, 2,2′‐(propane‐2,2‐diylbis(oxy) diethanamine, was synthesized using established procedures as previously described.^[^ ^33^ ^]^ In detail, *N*‐(2‐hydroxyethyl) phthalimde (5.1 g, 26.6 mmol, 1 equivalent) was dissolved in 50 mL of dry methylbenzene and cooled to 0°C using an ice bath (Figure S3A). Subsequently, 2‐methoxypropene (2.5 mL, 26.5 mmol, 1 equivalent) was slowly added to the solution in the presence of catalytic p‐toluenesulfonic acid (50.5 mg, 0.265 mmol, 0.01 equivalent). After the introduction of p‐toluenesulfonic acid, the reaction mixture quickly changed from a white suspension to an orange‐yellow clear solution. The reaction was stirred for 1 h at 0°C to prevent the loss of the highly volatile 2‐methoxypropene. Subsequently, the organic solvent was completely removed by vacuum evaporation. To quench the reaction, 50 mL of triethylamine was added, and unreacted alcohol groups were converted into the corresponding acetate by the addition of 1.5 mL of acetic anhydride. After overnight stirring at room temperature, the mixture was precipitated by slow addition to hexane. The precipitated powders were collected and underwent two recrystallizations from ethyl acetate, resulting in the formation of a faint yellow solid powder.

The deprotection of the synthesized 2,2′‐(propane‐2,2‐diylbis(oxy)bis(diethane‐2,1‐diyl) bis(isoindoline‐1,3‐dione) (1511.6 mg) was achieved by refluxing it overnight in 15 mL of 6 M NaOH to produce 2,2′‐(propane‐2,2‐diylbis(oxy))‐diethanamine. The final product was extracted three times using a mixed solvent of chloroform/isopropanol (1/1, v/v). The organic solvent was collected, dried with anhydrous sodium sulfate, and then evaporated under vacuum to yield a yellow oil. The structure of the product was verified using proton nuclear magnetic resonance (^1^H‐NMR).

### 4.9 Synthesis of DBCO‐DAK‐PEG‐NHS

DBCO‐NHS (10 mg, 0.0249 mmol, 1 equivalent) was dissolved in dry DCM at 10 mg mL^−1^, then DAK (4.04 mg, 0.0249 mmol, 1 equivalent) was added to the solution (Figure S3B). The solution was stirred for 8 h under nitrogen protection at room temperature. Meanwhile, COOH‐PEG‐COOH (124.5 mg, 0.0249 mmol, 1 equivalent) was dissolved in dry DCM with the addition of excess EDC and NHS (Figure S3B). After 8 h reaction, DBCO‐DAK solution was added drop by drop to NHS‐PEG‐NHS solution, and the reaction was stirred at room temperature in the dark for 24 h. The solution further underwent a 12 h dialysis process in ultrapure water, with regular replacements of ultrapure water every 4 h. Subsequently, the dialyzed solution was subjected to freeze‐drying, resulting in the formation of a white solid.

### 4.10 Synthesis of acid‐cleavable Tf (ACT)

Human holo‐Tf (10 mg, 0.000126 mmol, 1 equivalent) was dissolved in PBS containing 0.1 mM EDTA (pH 8.0) at 10 mg mL^−1^, and 5× molar excess DBCO‐DAK‐PEG‐NHS were introduced into the solution (Figure S3B). Next, an excess of EDC and NHS, each at a 2× molar ratio, was introduced into the solution and the solution was stirred overnight at room temperature under nitrogen protection. The solution further underwent a 12 h dialysis process in PBS, with regular replacements of ultrapure water every 4 h. This process ultimately yielded the desired orange ACT solution with a concentration of 10 mg mL^−1^.

### 4.11 Preparation of acid non‐cleavable Tf (DBCO‐PEG‐Tf or so‐called ANT): Synthesis of DBCO‐PEG‐NHS

DBCO‐NHS (10 mg, 0.0249 mmol, 1 equivalent) was dissolved in dry DCM at 10 mg mL^−1^. Meanwhile, 1 × molar NH_2_‐PEG‐COOH (49.8 mg, 0.0249 mmol, 1 equivalent) was dissolved in dry DCM with the addition of 2× molar excess EDC and NHS (vs NH_2_‐PEG‐COOH) (Figure S4A). After an 8 h reaction, DBCO‐NHS solution was added drop by drop to NH_2_‐PEG‐NHS solution, and the reaction was stirred at room temperature in the dark for 24 h. The solution underwent a 12 h dialysis process in ultrapure water, with regular replacements of ultrapure water every 4 h. Subsequently, the dialyzed solution was subjected to freeze‐drying, resulting in the formation of a white solid.

### 4.12 Synthesis of ANT

Human holo‐Tf (10 mg, 0.000126 mmol, 1 equivalent) was dissolved in PBS containing 0.1 mM EDTA (pH 8.0) at 10 mg mL^−1^, and 5× molar excess DBCO‐PEG‐NHS and 2× molar excess EDC and NHS were introduced into the solution. The solution was stirred overnight under nitrogen at room temperature. After that, the solution further underwent a 12 h dialysis process in PBS, with regular replacements of PBS every 4 h. This process ultimately yielded the desired orange ANT solution with a concentration of 10 mg mL^−1^.

### 4.13 Preparation of Ds@ACTE

The previously synthesized DBCO‐DAK‐PEG‐Tf was mixed with Exo‐N_3_ and incubated at 4°C overnight. The resulting mixture was subsequently subjected to dialysis to remove free DBCO‐DAK‐PEG‐Tf, yielding ACTE. To load siTGF‐β into ACTE, a solution containing 100 µg exosome was diluted in PBS containing 400 mM sucrose. Then, 100 µg of siTGF‐β and 200 µg of DOX‐HCl solution were introduced into the exosome solution, bringing the total volume to 400 µL, followed by a 10 min of incubation on ice. Electroporation was performed using a Gene Pulser Xcell electroporation system (Bio‐Rad electroporation system, USA) with a 4 mm‐gap cuvette, at 100 V and 125 µF. After electroporation, any aggregates formed during the process were gently dispersed, and the mixture was allowed to incubate at 37°C for 30 min to promote recovery of exosomal membranes. Ds@ACTE was obtained following the removal of excess siTGF‐β through ultracentrifugation. All experimental procedures were conducted under refrigerated conditions. Similarly, Ds@ANTE, Ds@PE, and Ds@Exo were prepared following the same protocol.

### 4.14 Western blot

Protein samples extracted from cells were separated or exosomes with sodium dodecyl sulfate‐polyacrylamide gel electrophoresis (SDS‐PAGE) and blotted onto the nitrocellulose (NC) membrane. The membrane was blocked with 5% skimmed milk, and incubated with an anti‐β‐actin antibody (1:1000; proteintech, 20536‐1‐AP), anti‐CD63 antibody (1:2000; proteintech, 67605‐1‐Ig) or anti‐TSG101 antibody (1:5000; proteintech, 67381‐1‐Ig) overnight at 4°C. After washing with TBST (Tris‐buffered saline with Tween20) three times, the membrane was further incubated with HRP‐conjugated anti‐mouse and anti‐rabbit secondary antibodies for 1 h at room temperature. The blots were imaged using a 5200 multiautomated chemiluminescence system (Tianneng, Shanghai, China).

### 4.15 In vitro cellular uptake

Following the instructions from the PKH‐26 labeling kit, a total of 1 × 10^10^ exosomes were diluted in diluent C to a final volume of 1 mL. Additionally, 6 µL of PKH26 or PKH67 solution (1 mg mL^−1^) was added to 1 mL of diluent C in another tube. These two solutions were mixed and incubated at room temperature in the dark for 5 min. The reaction was then terminated by adding 2 mL of 10% BSA‐containing PBS, bringing the final volume to 8.5 mL using PBS. Subsequently, 2 mL of 30% sucrose solution was added to the bottom of an ultracentrifuge tube to ensure that the PKH26‐labeled exosomes solution would be above the sucrose layer. The mixture was then ultracentrifuged at 120,000 × *g* for 90 min at 4°C. The entire solution, including the sucrose layer, was carefully aspirated, and the PKH26‐labeled exosomes were resuspended in 10 mL of cold PBS. Finally, the solution was centrifuged 3000 × *g* for 40 min at 4°C using an Amicon ultra‐centrifugal filter (10 kD MW cut‐off, Millipore Sigma, Missouri, USA) to remove the free dye.

Flow cytometry analysis was employed to assess cellular uptake by bEnd.3 cells. Briefly, bEnd.3 cells were seeded in 24‐well plates at 5 × 10^4^ cells per well and allowed growth for two days to reach approximately 80% cell confluence. The culture medium was then removed and replaced with fresh FBS‐free medium containing PBS and PKH26‐labeled Exo, PE, ANTE, and ACTE (5 × 10^9^ particles per well). After 2, 4, and 8 h of incubation, cells were digested, collected, washed twice with PBS, and then resuspended in 500 µL of PBS for flow cytometric analysis (BD, USA).

For CLSM observation, bEnd.3 cells were seeded in 6‐well plates pre‐placed with glass coverslips of 400 mm^2^ at 5 × 10^4^ cells per well. The cells were allowed to grow for 24 h to reach approximately 80% confluence. Then, the culture medium was replaced with fresh FBS‐free medium containing PBS and PKH67‐labeled Exo, PE, ANTE, and ACTE (5 × 10^9^ particles per well). After 1, 4, and 24 h of incubation, Lysotracker Red was added to the medium at a 1:7000 ratio at 30 min before the end of incubation. Following the experimental protocol, the cells were washed twice with PBS, fixed with a 4% PFA solution for 15 min, and underwent an additional round of PBS washing. Afterward, the cells were stained with DAPI for 5 min, transferred to a carrier slide, and sealed with 20 µL of anti‐fade mounting medium. Finally, the fluorescence signal was observed using CLSM (N‐SIM E, Nikon, Japan).

### 4.16 Uptake mechanism

To elucidate the underlying mechanism involved in the cellular uptake of ACTE, various endocytic inhibitors, including methyl‐β‐cyclodextrin (M‐β‐CD, 5 mM), amiloride (Ami, 100 µM), chlorpromazine (Chlo, 30 µM), genistein (Geni, 1 mM), and free Tf (100 µg mL^−1^), were pre‐incubated with bEnd.3 cells for 0.5 h, followed by incubation with PKH26‐labeled ACTE. Cells without treatment served as the positive control. After 4 h of incubation, cellular uptake was conducted using flow cytometry.

### 4.17 Transcytosis evaluation using in vitro BBB model

In vitro BBB model was established using Millicell hanging cell culture inserts. In brief, bEnd.3 cells were seeded at a density of 8 × 10^4^ into the Transwell upper chambers pre‐inserted into a 6‐well plate. The cells were cultured continuously for 4 days with medium changes during this period. The transendothelial electrical resistance (TEER) of the bEnd.3 monolayer was measured using Millicell ERS (Millipore, USA) until it exceeded 200 Ω, indicating the formation of tight junctions. Before the completion of the bEnd.3 monolayer, GL261 cells were seeded into another 6‐well plate pre‐placed with glass coverslips of 400 mm^2^. The culture medium in the upper chamber was replaced with PBS containing PKH67‐labeled Exo, PE, ANTE, and ACTE (5 × 10^9^ particles per well) and transferred to the 6‐well plate containing GL261 cells. Following the experimental procedure, cells underwent two PBS washes, were fixed with 4% paraformaldehyde for 15 min, and underwent another round of PBS washing. Subsequently, the cells were stained with DAPI for 5 min, transferred onto a carrier slide, and sealed using 20 µL of anti‐fade mounting medium. Meanwhile, the bEnd.3 monolayer was removed, blocked with TBS containing 2% goat serum for 1 h, and then incubated with rabbit anti‐mouse ZO‐1 antibody (1:200, proteintech; 21773‐1‐AP). Afterward, Cy3‐conjugated goat anti‐rabbit antibody was further introduced for immunofluorescence staining and the fluorescence imaging was observed using CLSM.

### 4.18 Quantitative evaluation of transcytosis using flow cytometry

According to the method described above, an in vitro BBB model using a 12‐well plate was further constructed. The TEER of the bEnd.3 monolayer was ensured to exceed 200 Ω before proceeding with subsequent investigations. GL261 cells were seeded at a density of 5 × 10^4^ in the bottom 12‐well plate and allowed to grow for 24 h. Before the completion of the bEnd.3 monolayer, GL261 cells were seeded into another 12‐well plate. The culture medium in the upper chamber was replaced with PBS containing PKH26‐labeled Exo, PE, ANTE, and ACTE (5 × 10^9^ particles per well) and transferred to the 12‐well plate containing GL261 cells. Following 6 h of co‐culture, the upper bEnd.3 cells and lower GL261 cells were harvested separately, washed twice with PBS, and finally resuspended in 0.3 mL of PBS for subsequent flow cytometry analysis.

### 4.19 Evaluation of apoptosis‐induced ability using in vitro BBB model

The apoptosis‐induced ability of Ds@ACTE on GL261 cells was analyzed using the Annexin V‐FITC/PI apoptosis detection kit (BD, USA). The 12‐well plate BBB model was constructed following the procedure described above. GL261 cells were seeded in 12‐well plates at a density of 5 × 10^4^ cells per well. After 24 h of culture, Ds, Ds@Exo, Ds@PE, Ds@ANTE, and Ds@ACTE were added to the upper bEnd.3 cell layer and co‐cultured for 24 h, with a siTGF‐β dosage of 200 nM and 1 µg mL^−1^ of DOX. Cells cultured in DMEM medium containing 10% FBS served as the negative control. Subsequent staining procedures were conducted according to the manufacturer's instructions. Annexin V‐FITC/PI double staining was analyzed using flow cytometry (FACSverse, BD), and data were analyzed using Flowjo V10 software.

### 4.20 RT‐qPCR analysis

To assess the gene inhibition efficiency of siTGF‐β in the in vitro BBB model, a 6‐well plate BBB model was established following the previously described methods. GL261 cells were seeded into the 6‐well plate at a density of 2 × 10^5^ cells per well. After overnight incubation, cells were treated with Ds@ACTE at a siTGF‐β concentration of 200 nM. siTGF‐β‐loaded Lipo2000 was used as a positive control and GL261 cells without treatments served as negative control. Following 24 h of incubation, total RNA was purified using Trizol (Invitrogen) according to the manufacturer's protocol. Total RNA was then reverse transcribed using MultiScribe reverse transcriptase and oligo(dT) primers (Table S3). Quantitative PCR (qPCR) analysis was performed using Hieff qPCR SYBR Green Master Mix on an ABI QuantStudio 3 system. β‐actin was used as the housekeeping gene. Each reaction included three technical replicates, and the average was considered one biological replicate. The experiment was repeated three times on different days, with each experiment defining a biological replication. Statistical analysis was conducted on the Δ*Ct* values of biological replicates (mice or independent experiments). Results were normalized to the relative expression level of the negative control group.

### 4.21 In vivo living imaging

GL261 glioma‐bearing mice were established according to the previously published method.^[^ ^22b^ ^]^ In brief, male BALB/c mice were anesthetized using Chloralhydrate. GL261 cells (2.5 × 10^5^ cells suspended in 5 µL PBS) were implanted into the right striatum of the mice using a brain stereotactic fixation device (lateral 1.8 mm, anteroposterior 0.6 mm, depth 3 mm) with a mouse adapter. Two weeks after implantation, the intracranial GL261 glioma mice were randomly divided into five groups (3 mice per group): PBS, Exo, PE, ANTE, and ACTE. Before injection, we labeled exosomes with the near‐infrared lipophilic carbocyanine dye DiD (Invitrogen, USA) following a protocol similar to PKH26 labeling. Briefly, 5 µL of DiD (1 mg mL^−1^ in ethanol) was mixed with 5 × 10^10^ exosomes in 1 mL PBS and incubated for 20 min at room temperature. Subsequently, we performed spin column purification following the previously outlined PKH‐67 labeling protocol to eliminate ethanol and unbound DiD. To be specific, a total of 1 × 10^10^ exosomes were diluted in diluent C to a final volume of 1 mL. Additionally, 6 µL of DiD solution (1 mg mL^−1^) was added to 1 mL of PBS in another tube. These two solutions were mixed and incubated at room temperature in the dark for 30 min. The reaction was then terminated by adding 2 mL of 10% BSA‐containing PBS, bringing the final volume to 8.5 mL using PBS. Subsequently, 2 mL of 30% sucrose solution was added to the bottom of an ultracentrifuge tube to ensure that the DiD‐labeled exosomes solution would be above the sucrose layer. The mixture was then ultracentrifuged at 120,000 × *g* for 90 min at 4°C. The entire solution, including the sucrose layer, was carefully aspirated, and the DiD‐labeled exosomes were resuspended in 10 mL of cold PBS. Finally, the solution was centrifuged 3000 × *g* for 40 min at 4°C using an Amicon ultra‐centrifugal filter (10 kD MW cut‐off, Millipore Sigma, Missouri, USA) to remove the free dye. DiD‐labeled exosomes were then administered to GL261‐bearing mice via intravenous injection at a dosage of 1 × 10^10^ exosomes (≈0.15 mL) per mouse. The whole‐body fluorescence biodistribution of these labeled exosomes at 0.5, 1, 2, 4, 8, and 24 h was monitored using the IVIS Lumina II optical imaging system (Xenogen, Caliper Life Sciences, USA). During the imaging process, mice were pre‐anesthetized using 2%–4% isoflurane in 100% oxygen. After the last living imaging at 24 h, the mice were euthanized and their major organs (brain, heart, liver, lung, kidney, spleen) were subjected to ex vivo imaging. Imaging conditions encompassed the utilization of a filter set for fluorescence imaging. The scan parameters were set as follows: excitation = 644 nm, emission = 665 nm, field of view = 13.5 cm, f‐stop = 2, light fluence = 2 mW cm^−2^. Camera settings were configured for maximum gain, a binning factor of 4, and an exposure time of 4 seconds. Data analysis was performed using IVIS software.

### 4.22 Immunofluorescence staining

The brain and other organs were initially fixed with 4% paraformaldehyde for 24 h. Subsequently, these organs underwent a gradient dehydration in 15% and 30% sucrose solutions for 24 h, respectively. After washing, permeabilization, and blocking frozen brain sections were stained with anti‐TfR antibody (at a dilution of 1:100) and anti‐integrin αv antibody (at a dilution of 1:500) overnight at 4°C, followed by incubation with the corresponding FITC‐conjugated secondary antibody (at a dilution of 1:200) for 2 h at room temperature. Throughout all experimental procedures, dilution and washing were performed using TBS. Analysis was carried out using a CLSM (N‐SIM E, Nikon, Japan).

### 4.23 In vivo anti‐GBM evaluation

To investigate the anti‐GBM effects of different formulations, the GL261 GBM‐bearing C57BL6/j mice model was established following the methods described above. Seven days post‐implantation, the mice were randomly divided into six groups (13 mice per group). On days 8, 10, 12, and 14, each group of mice received intravenous injections of PBS, Ds, Ds@Exo, Ds@PE, Ds@ANTE, and Ds@ACTE. The administration dose of DOX and siTGF‐β was at an equivalent dose of 1 mg kg^−1^ and 1 mg kg^−1^, respectively. For all groups, the overall survival and body weight were monitored pre‐ and post‐administration using 10 mice per group. Survival time was calculated from the day of GL261 cell implantation (day 0) until the day of death. Kaplan–Meier survival curves were generated for each group.

One day after the final administration (Day 15), three mice from each group were randomly selected and euthanized. Subsequently, brain, heart, liver, spleen, lung, and kidney were collected for hematoxylin and Eosin (H&E) staining. Brains were further sampled for immunohistochemical (IHC) staining of Ki67, CD8, CD4, and FoxP3. In addition, blood samples were collected for serum biochemical analysis. Spleen and lymph nodes were isolated for immunological analysis.

Spleens and lymph nodes from different groups of mice were isolated and single‐cell suspensions were prepared. Briefly, spleens and lymph nodes were ground in fresh RPMI‐1640 medium and filtered through a 40‐µm cell strainer (Fisher Scientific). The resulting cells were washed with PBS, lysed with red blood cell lysis buffer, and blocked with PBS solution containing 2% BSA. Cells were then labeled with fluorescent antibodies following standard protocols. To study regulatory T cells (Tregs), anti‐mouse APC‐CD3 antibody (1:100), lymphocytes, and splenocytes were stained with anti‐mouse FITC‐CD4 antibody (1:200), and anti‐mouse PE‐FoxP3 antibody (1:200) were used for staining. For the analysis of cytotoxic T lymphocytes (CTLs), cells were stained with anti‐mouse APC‐CD3 antibody (1:100), anti‐mouse Pacific Blue‐CD8 antibody (1:200), and anti‐mouse FITC‐CD4 antibody (1:200) according to standard procedures. To analyze natural killer (NK) cells (CD3^−^CD49b^+^), lymphocytes were co‐stained with anti‐mouse APC‐CD3 antibody (1:100) and anti‐mouse eFluor 450‐CD49b antibody (1:200).

### 4.24 Statistical analysis

Data was processed by GraphPad Prism 9.5.0 and Origin 2021. Unless otherwise specified, all data are presented as mean ± standard deviation (SD). Statistical significance was assessed using one‐way and two‐way analysis of variance (ANOVA) turkey. Survival was determined using Kaplan–Meier survival plot and survival analysis was calculated using log‐rank (Mantel–Cox) test (SPSS 16.0). Differences are labeled ns (no significance) for not significant, * for *p *< 0.05, ** for *p* < 0.01, *** for *p* < 0.001, and **** for *p* < 0.0001.

## AUTHOR CONTRIBUTIONS

Shaobo Ruan, Yuhua Weng conceived and designed the project. Jun Yang, Yong Li, Shaoping Jiang, Yuxin Tian, Mengjie Zhang, Shuai Guo, Pengfei Wu, Jianan Li, Lin Xu, Wenpei Li, Yushu Wang, Huile Gao, Yuanyu Huang processed the experiments and collected the data. Shaobo Ruan, Yuhua Weng, Jun Yang, and Yong Li analyzed the data, drafted the manuscript, and revised the manuscript.

## CONFLICT OF INTEREST STATEMENT

The authors declare no competing financial interest or personal relationships that could have appeared to influence the work reported in this paper. Yuanyu Huang is a member of the *Exploration* editorial board.

## Supporting information

Supporting Information

## Data Availability

Data will be made available on request.
