# Erratum to “Engineered Brain‐Targeting Exosome for Reprogramming Immunosuppressive Microenvironment of Glioblastoma”

**DOI:** 10.1002/EXP.70002

**Published:** 2025-02-04

Jun Yang^1,#^, Yong Li^1,#^, Shaoping Jiang^1^, Yuxin Tian^1^, Mengjie Zhang^1^, Shuai Guo^1^, Pengfei Wu^1^, Jianan Li^1^, Lin Xu^1^, Wenpei Li^1^, Yushu Wang^2^, Huile Gao^3^, Yuanyu Huang^1^, Yuhua Weng^1,*^, Shaobo Ruan^1,*^

^1^School of Life Science, Advanced Research Institute of Multidisciplinary Science, Laboratory of Molecular Medicine and Biotherapy, Beijing Institute of Technology, Beijing, China

^2^Department of Biomedical Engineering, Tufts University, Medford, Massachusetts, USA

^3^West China School of Pharmacy, Sichuan University, Chengdu, China

J. Yang, Y. Li, S. Jiang, Y. Tian, M. Zhang, S. Guo, P. Wu, J. Li, L. Xu, W. Li, Y. Wang, H. Gao, Y. Huang, Y. Weng, S. Ruan, *Exploration* 2024, 20240039.

In our manuscript titled “Engineered brain‐targeting exosome for reprogramming immunosuppressive microenvironment of glioblastoma,” the in vivo living images of G4 group at 0.5 and 1 h in Figure 5A were misplaced, and the in vivo living image of G3 group at 2 h used the wrong mouse due to a formatting error. The error was spotted in time after revisiting the article and we corrected this error by providing the revised image.

**FIGURE 5:**
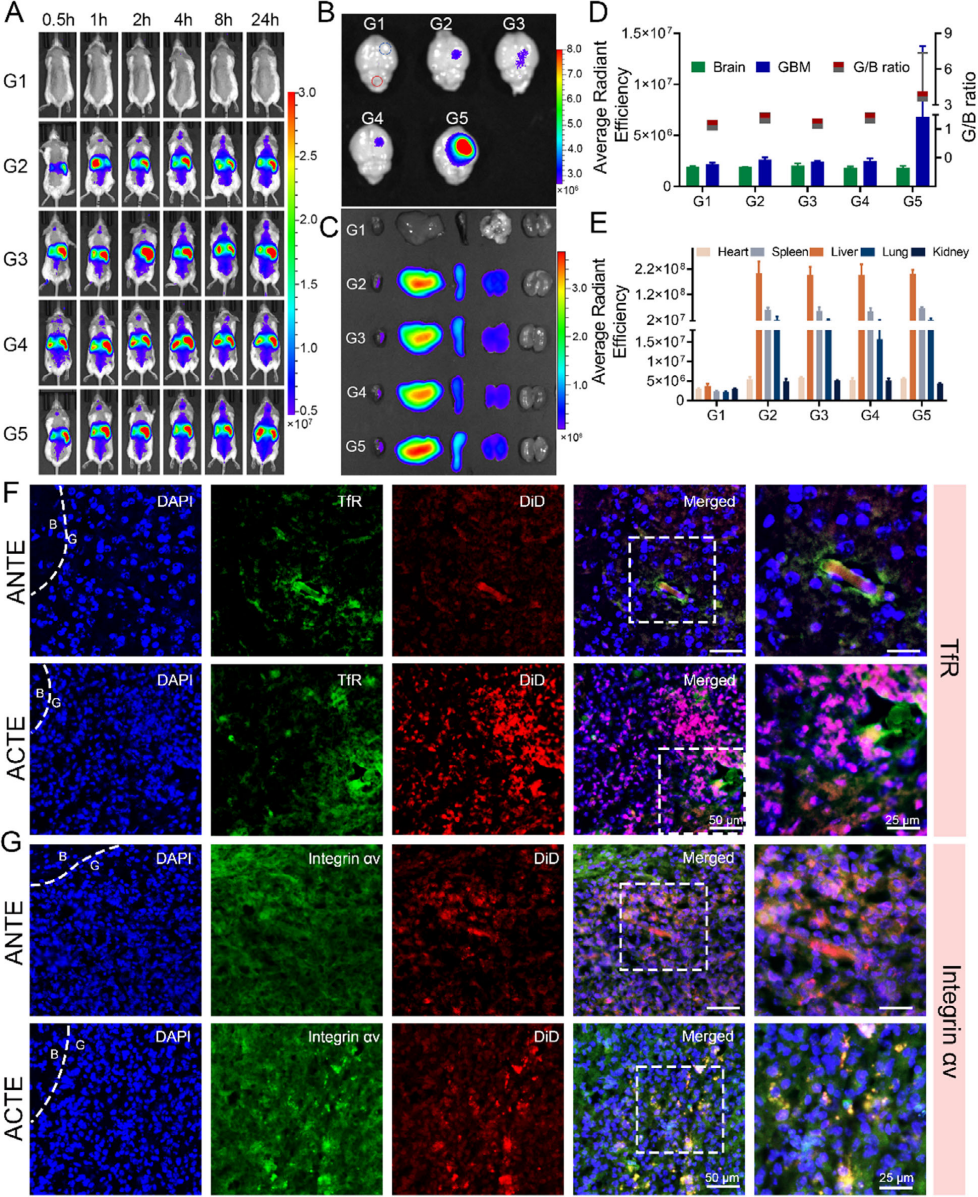
Evaluation of in vivo distribution and GBM‐targeting efficiency of ACTE. (A) Living images of GL261‐bearing mice after intravenous injection with ACTE and control‐engineered exosomes at different time intervals. G1 to G5 represents PBS, Exo, PE, ANTE and ACTE, bar represents radiant efficiency from 5.0 × 10^6^ to 3.0 × 10^7^ [p s^−1^ cm^−2^ sr^−1^]/[µW cm^−2^]. (B) Ex vivo imaging of brains collected at 24 h after injection, the blue circle represents the glioma site, the red circle represents normal brain parenchyma, bar represents radiant efficiency from 2.7 × 10^6^ to 8.0 × 10^6^ [p s^−1^ cm^−2^ sr^−1^]/[µW cm^−2^]. (C) Ex vivo imaging of major organs collected at 24 h after injection, bar represents radiant efficiency from 1.0 × 10^7^ to 3.0 × 10^8^ [p s^−1^ cm^−2^ sr^−1^]/[µW cm^−2^]. (D) The left column represents the semi‐quantitative date of the fluorescence signal at the indicative site from B, right column represents the *G*/*B* ratio of different exosomes (*n* = 3). (E) Semi‐quantitative data of the fluorescence signal of major organs from (C) (*n* = 3). (F) Fluorescence distribution of ANTE and ACTE at glioma site after intravenous administration for 24 h, GBM slices were immune‐stained with anti‐TfR antibody. Scale bar is 50 µm (left) and 25 µm (right). (G) Fluorescence distribution of ANTE and ACTE at glioma site after intravenous administration for 24 h, GBM slices were immune‐stained with anti‐integrin αv antibody. Scale bar is 50 µm (left) and 25 µm (right).

We apologize for this error.

