# Supplementary material for: Engineered brain‐targeting exosome for reprogramming immunosuppressive microenvironment of glioblastoma
Source: Exploration (Beijing). 2024 Jun 26;5(2):20240039. doi: 10.1002/EXP.20240039 (PMC12087404; doi:10.1002/EXP.20240039)
Supplement: Supplementary file 1 — Supporting Information [file EXP2-5-20240039-s001.pdf]

Supporting Information

Engineered Brain-Targeting Exosome for Reprogramming Immunosuppressive Microenvironment of Glioblastoma

Jun Yang[1,#], Yong Li[1,#], Shaoping Jiang[1], Yuxin Tian[1], Mengjie Zhang[1], Shuai Guo[1], Pengfei Wu[1], Jianan Li[1], Lin Xu[1], Wenpei Li[1], Yushu Wang[2], Huile Gao[3], Yuanyu Huang[1], Yuhua Weng[1,*], Shaobo Ruan [1,*]

[1] School of Life Science, Advanced Research Institute of Multidisciplinary Science, Laboratory of Molecular Medicine and Biotherapy, Beijing Institute of Technology, Beijing, 100081, China.

[2] Department of Biomedical Engineering, Tufts University, 4 Colby Street, Medford, MA 02155, USA

[3] West China School of Pharmacy, Sichuan University, Chengdu, 610041, China.

# These authors contributed equally.
*Corresponding authors: ruanshaobo@bit.edu.cn; wengyh@bit.edu.cn

Keywords: Engineered exosomes, Glioblastoma, RNA interference, Immunosuppressive microenvironment, Chemo-resistance

Supplementary Tables

Table S1. Median survival and mean survival of GL261 GBM-bearing mice treated with different formulations (n = 10).

Group	Mean (day)	Median (day)	Standard error (day)	Increased median survival time
Ds@ACTE	40.7	39	1.6	70%
Ds@ANTE	33.3	34	3.2	48%
Ds@PE	27.4	26	1.6	13%
Ds@Exo	27.4	26	2.4	13%
DOX&siTGF-β	27.9	28	3.2	22%
PBS	23.2	23	1.6	

Table S2. Pairwise comparison for survival using log-rank (Mantel-Cox) test (n = 10).

	1		2		3		4		5		6	
	a	b	a	b	a	b	a	b	a	b	a	b
1			6.753752	0.009355	3.433556	0.063884	5.185159	0.022781	16.32612	5.33E-05	17.98744	2.22E-05
2	6.753752	0.009355			0.005445	0.941175	0.250611	0.616645	8.764889	0.003071	12.94267	0.000321
3	3.433556	0.063884	0.005445	0.941175			0.002221	0.962411	4.698739	0.030185	13.02485	0.000307
4	5.185159	0.022781	0.250611	0.616645	0.002221	0.962411			7.212711	0.007239	13.63272	0.000222
5	16.32612	5.33E-05	8.764889	0.003071	4.698739	0.030185	7.212711	0.007239			7.520127	0.006101
6	17.98744	2.22E-05	12.94267	0.000321	13.02485	0.000307	13.63272	0.000222	7.520127	0.006101		

1 represents PBS, 2 represents DOX&siTGF-β, 3 represents Ds@Exo, 4 represents Ds@PE, 5 represents Ds@ANTE, 6 represents Ds@ACTE, a represents Chi-square, b represents significance.

Table S3. Primer's information

Primer	Sequence (5′-3′)
β-actin	F: AGCCATGTACGTAGCCATCC
	R: CTCTCAGCTGTGGTGGTGAA
siTGF-β	F: CTGATACGCCTGAGTGGCTG
	R: TTTGGGGCTGATCCCGTTG

Supporting Figures

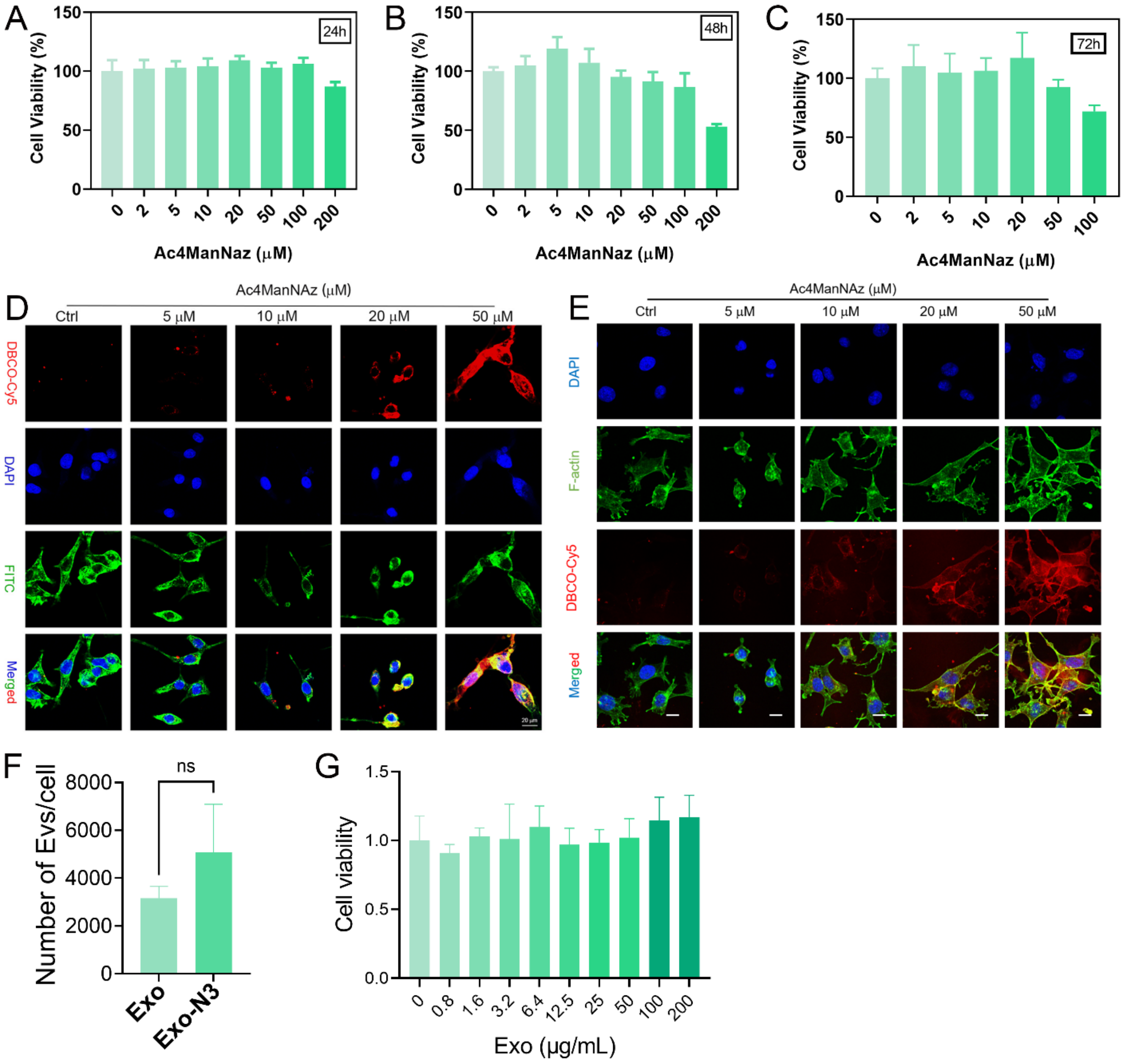

Figure S1. (A, B, C) The cytotoxicity of different concentrations of Ac4ManNAz in GL261 cells at 24 h (A), 48 h (B), and 72 h (C). (D, E) CLSM images of DBCO-Cy5 labeled GL261 cells after pre-treatment with Ac4ManNAz (5-50 μM) for 24 h (D) and 72 h (E). Scale bar is 20 μm. (F) The average cellular secretion rate for exosomes production from both GL261 and Ac4ManNAz-treaded GL261. (G) Cell viability of bEnd.3 cells after treatment with GL261-derived exosomes at different concentrations.

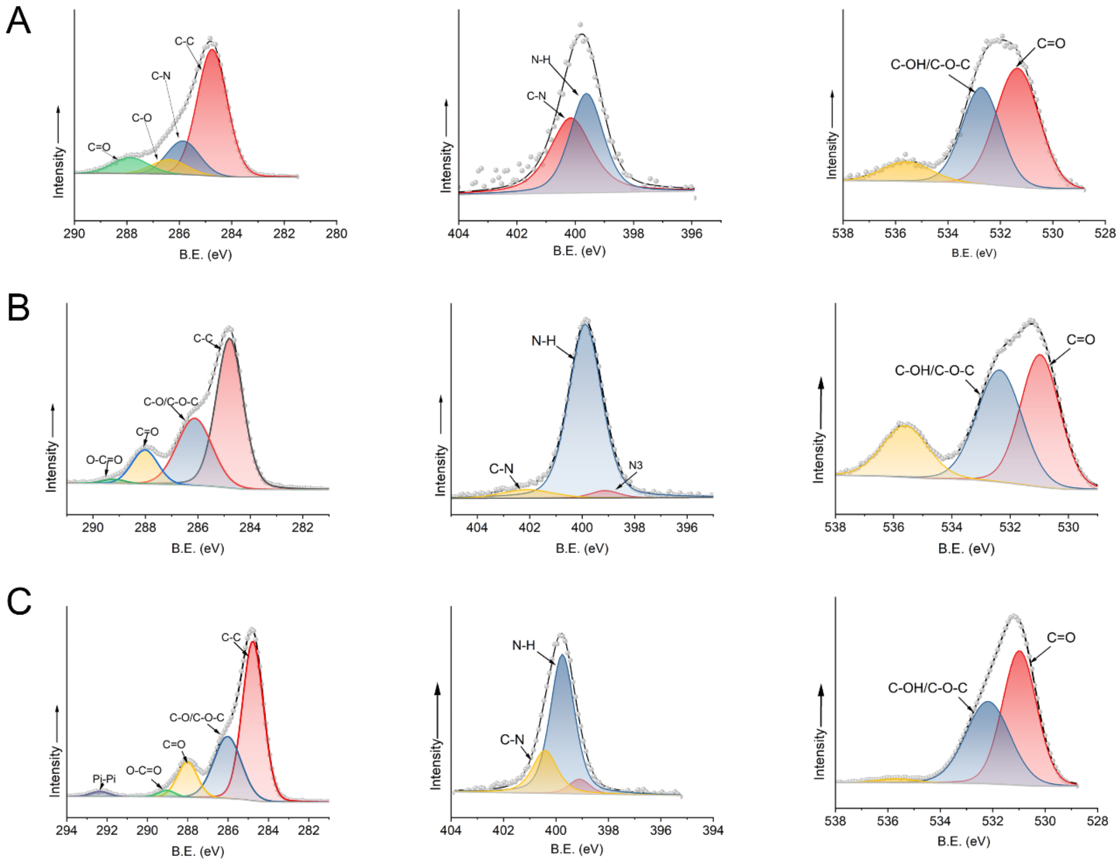

Figure S2. (A) XPS C_{1s}, O_{1s}, and N_{1s} spectrum of Exo, respectively. (B) XPS C_{1s}, O_{1s}, and N_{1s} spectrum of Exo-N_3, respectively. (C) XPS C_{1s}, O_{1s}, and N_{1s} spectrum of Exo-DBCO, respectively.

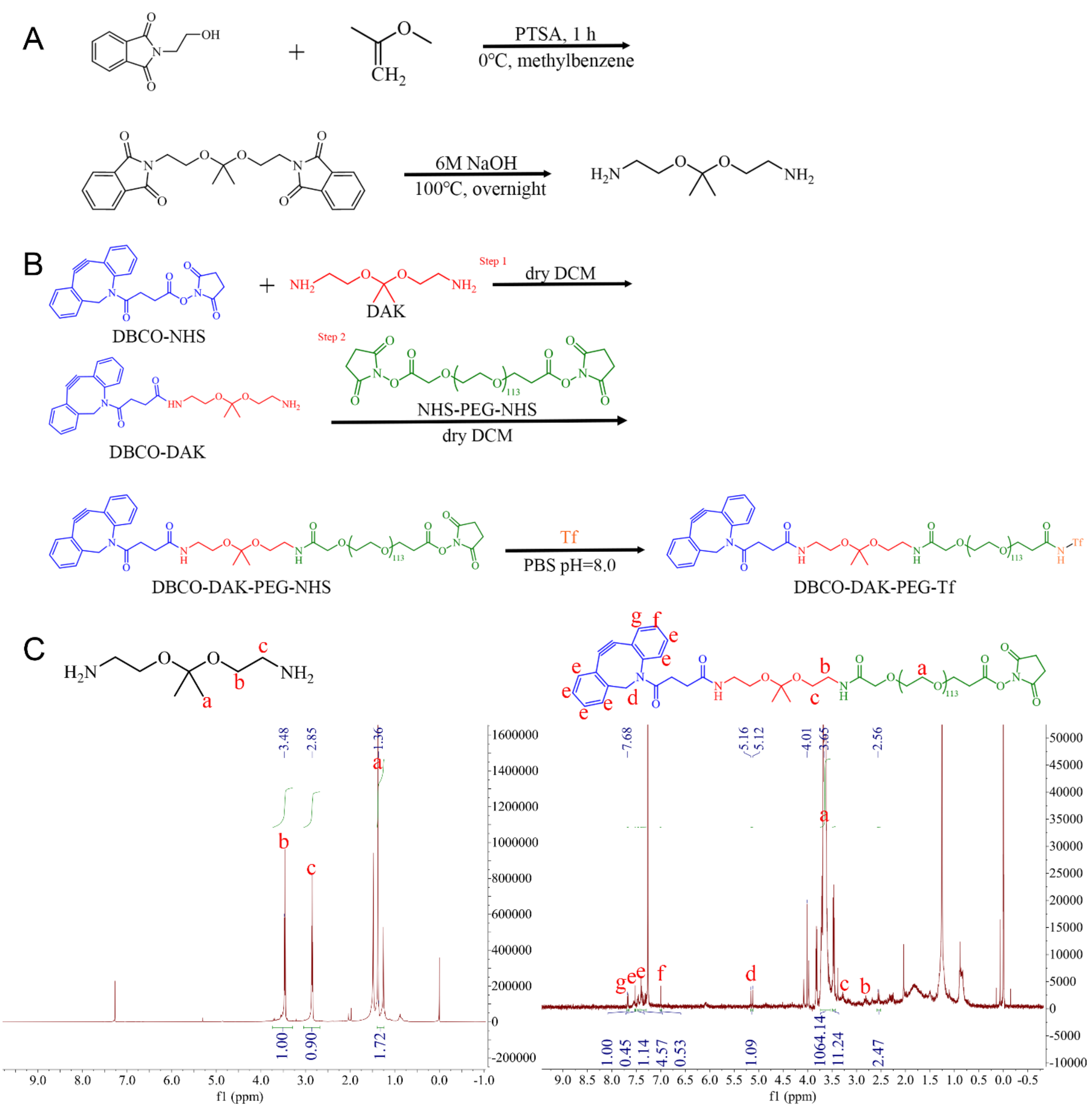

Figure S3. (A) Synthetic routes of acid-liable DAK. (B) Synthetic routes of DBCO-DAK-PEG-NHS. (C) ^1H NMR spectrum of DAK and DBCO-DAK-PEG-NHS in deuterated chloroform.

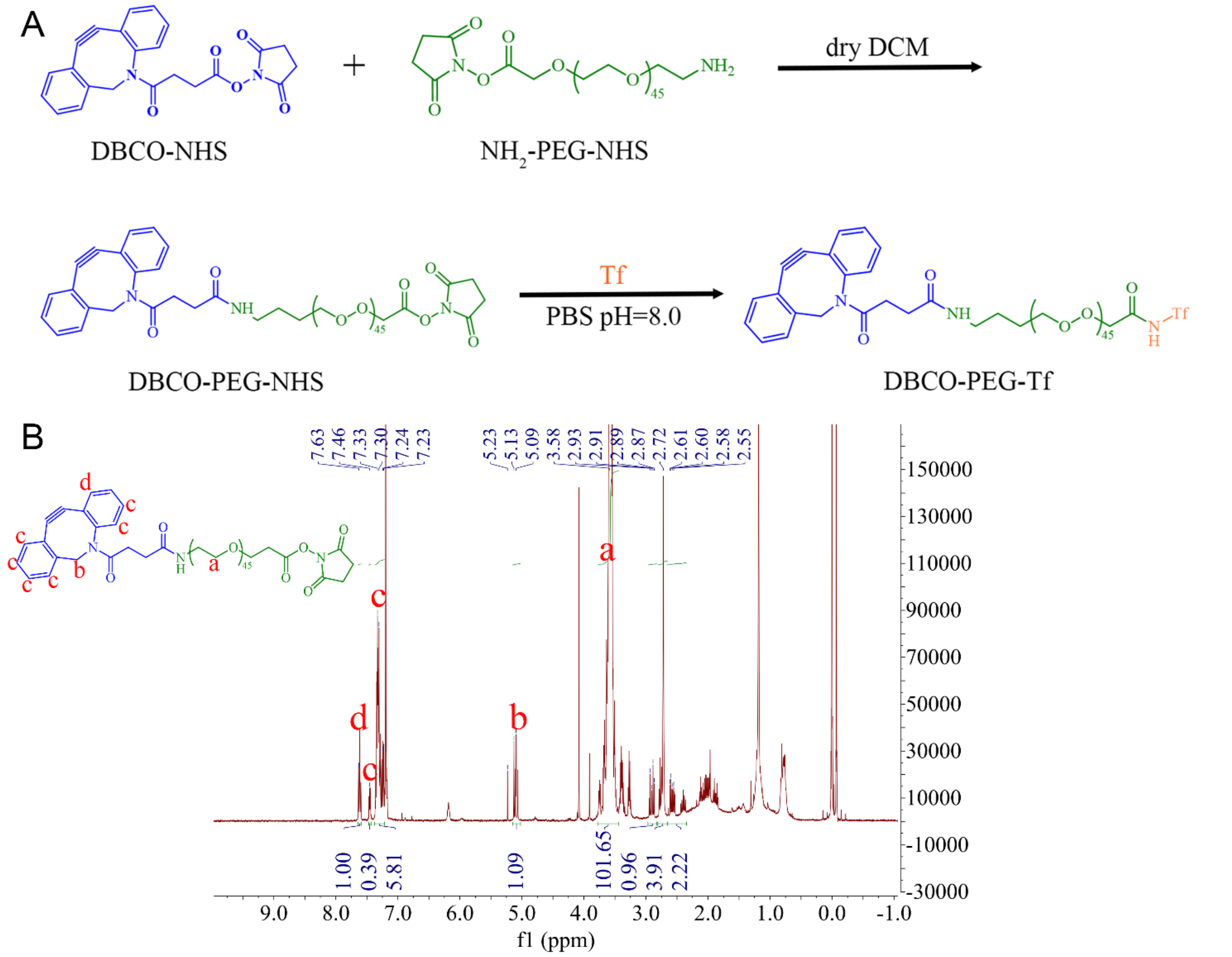

Figure S4. (A) Synthetic routes of acid non-cleavable Tf. (B) ^{1}H NMR spectrum of DBCO-PEG-NHS in deuterated chloroform.

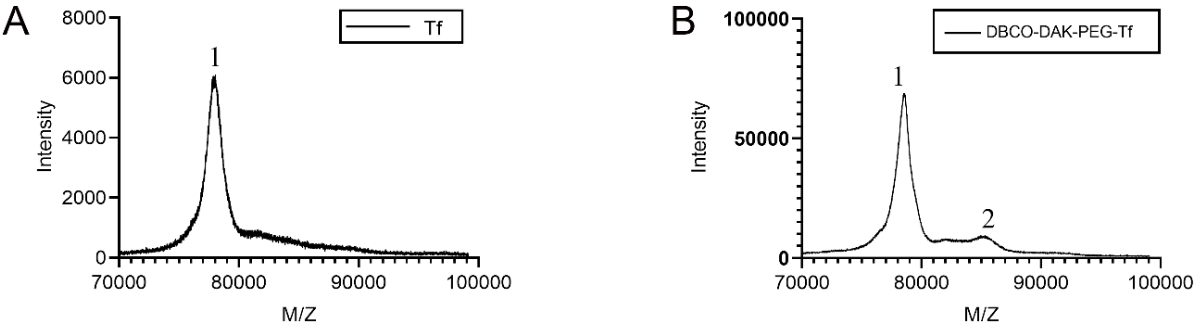

Figure S5. Matrix-assisted laser desorption/ionization-time of flight (MALDI-TOF) spectra of purified conjugates. (A) MALDI-TOF spectrum of purified Tf (approximately 80 kDa, peak 1). (B) MALDI-TOF spectrum of DBCO-DAK-PEG-Tf (approximately 85 kDa, peak 2).

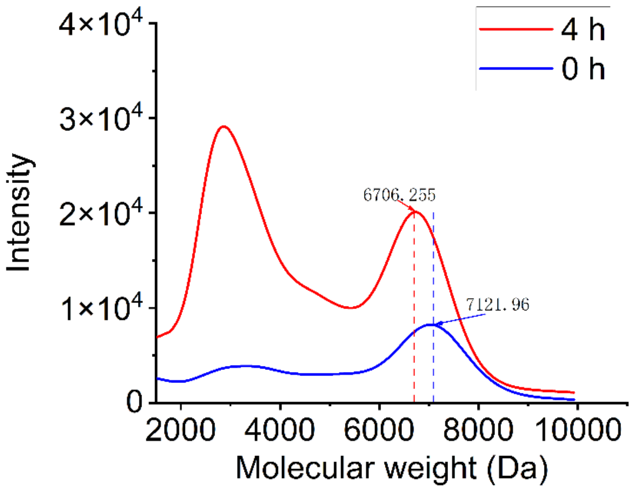

Figure S6. MALDI-TOF spectrum of DBCO-DAK-PEG over time in pH 5.0 PBS at 37°C, which indicated the pH-cleavable ability of DAK.

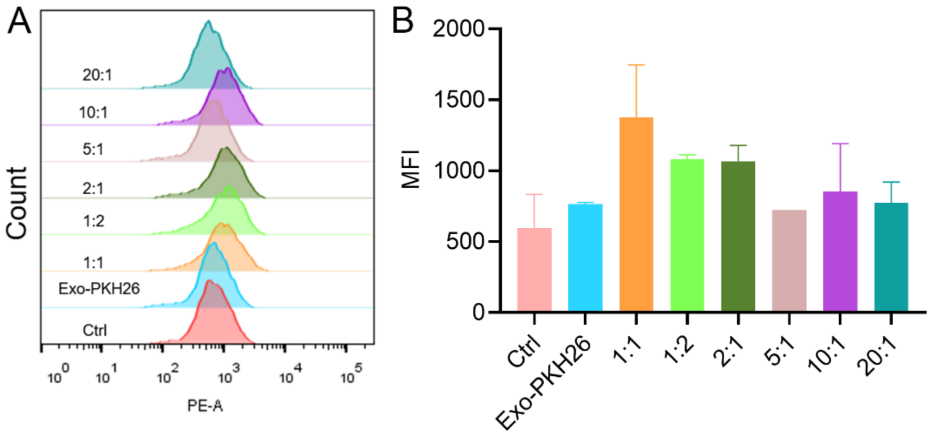

Figure S7. (A, B) Uptake of ACTE in bEnd.3 cells at different ratios (Exo-N_3: ACT).

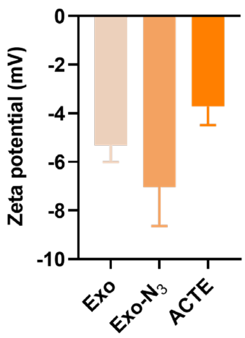

Figure S8. Zeta potential results of Exo, Exo-N_3, and ACTE.

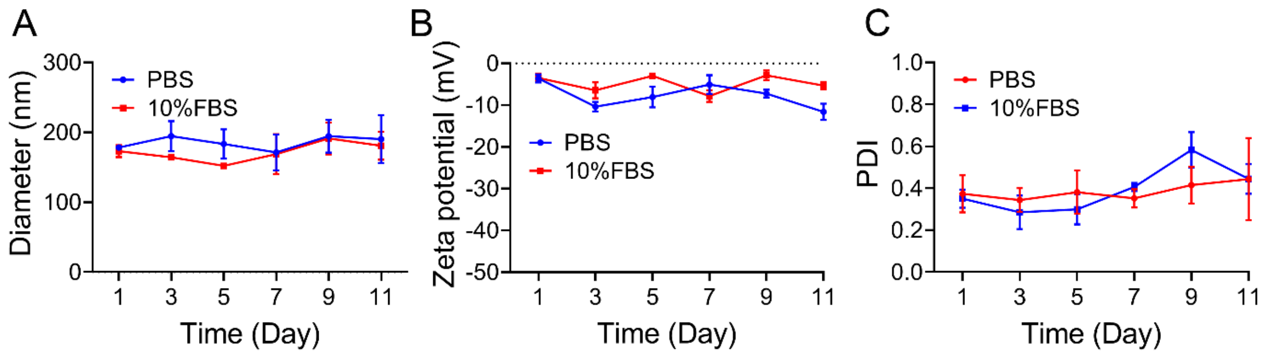

Figure S9. Stability of ACTE. (A) The particle size of ACTE in PBS and 10% FBS at 4°C. (B) The zeta potential of ACTE in PBS and 10% FBS at 4°C. (C) The polydispersity index (PDI) of ACTE in PBS and 10% FBS at 4°C.

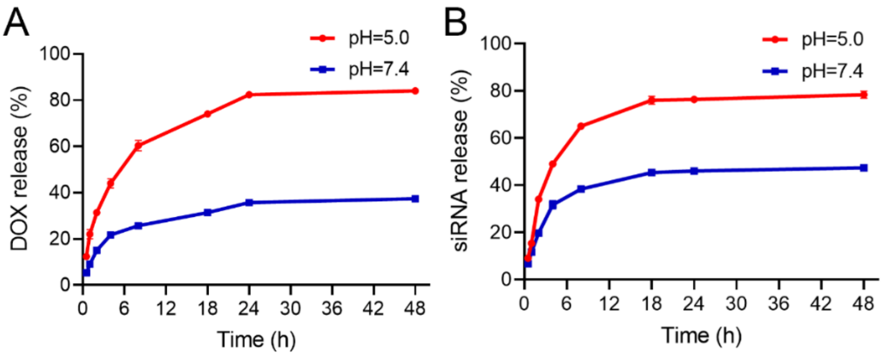

Figure S10. The release profile of DOX (A) and siRNA (B) in pH 7.4 and 5.0 PBS.

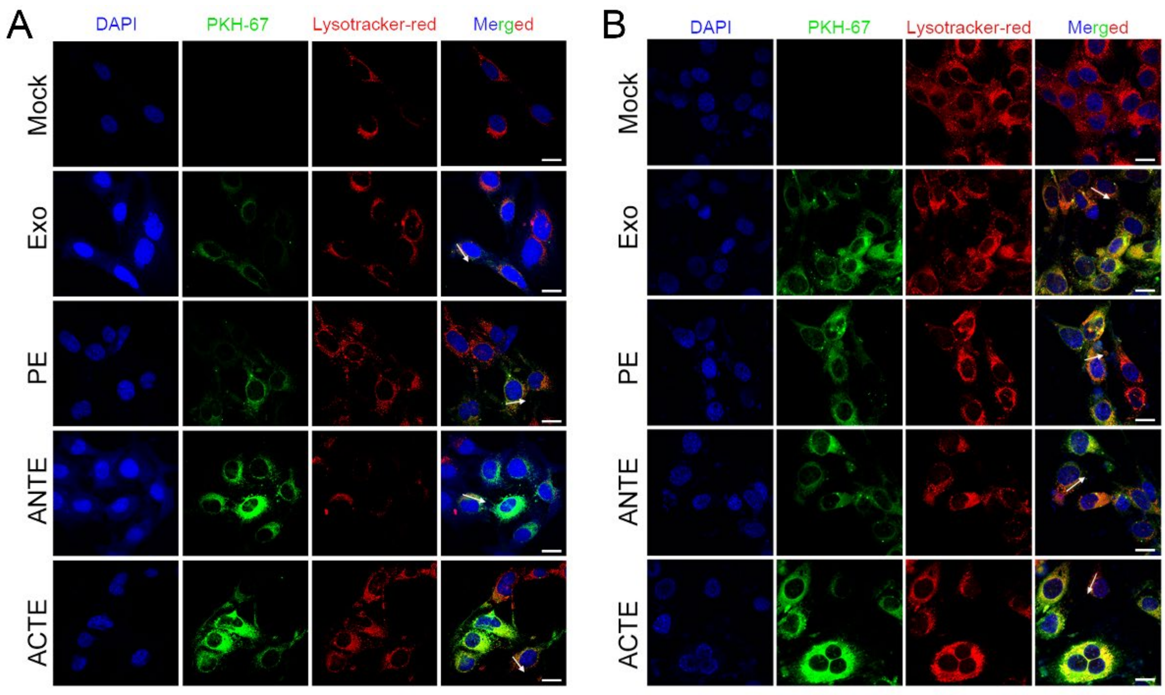

Figure S11. (A, B) Confocal images of bEnd.3 cells were treated with different formulations for 1 h (A) and 24 h (B). Scale bar is 20 μm.

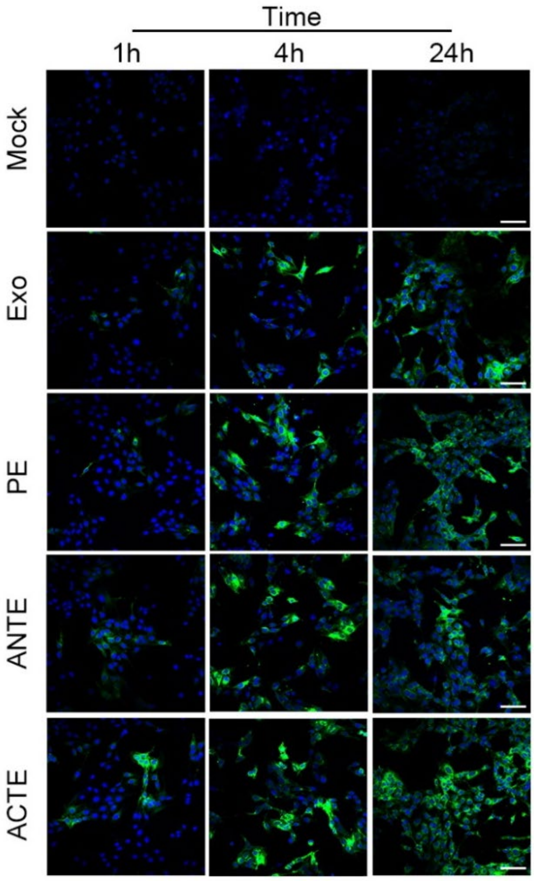

Figure S12. Confocal images of bEnd.3 cells were treated with different formulations for different time. Scale bar is 100 μm.

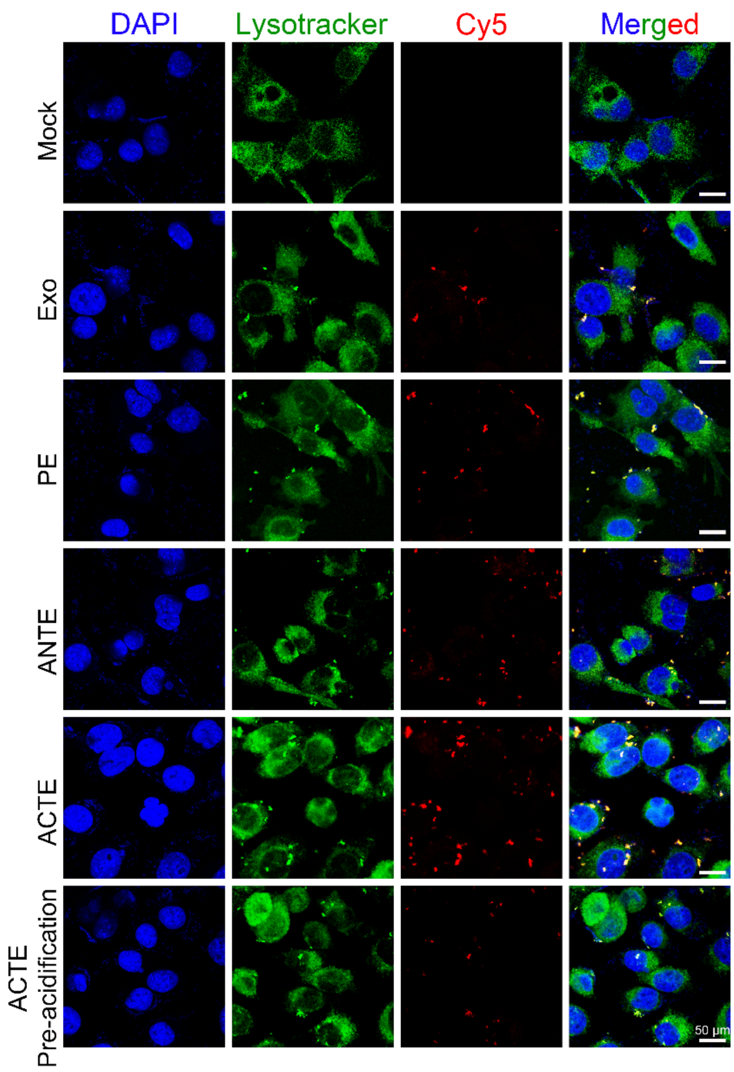

Figure S13. Representative CLSM images of GL261 cells after incubation of different engineered exosomes for 4 h. Scare bar is 50 μm.

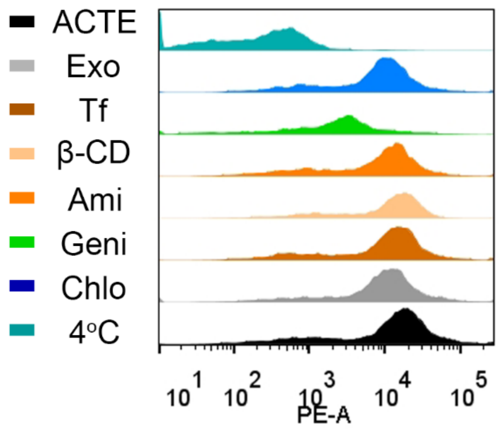

Figure S14. Flow cytometry plots of treated cells with different inhibitors.

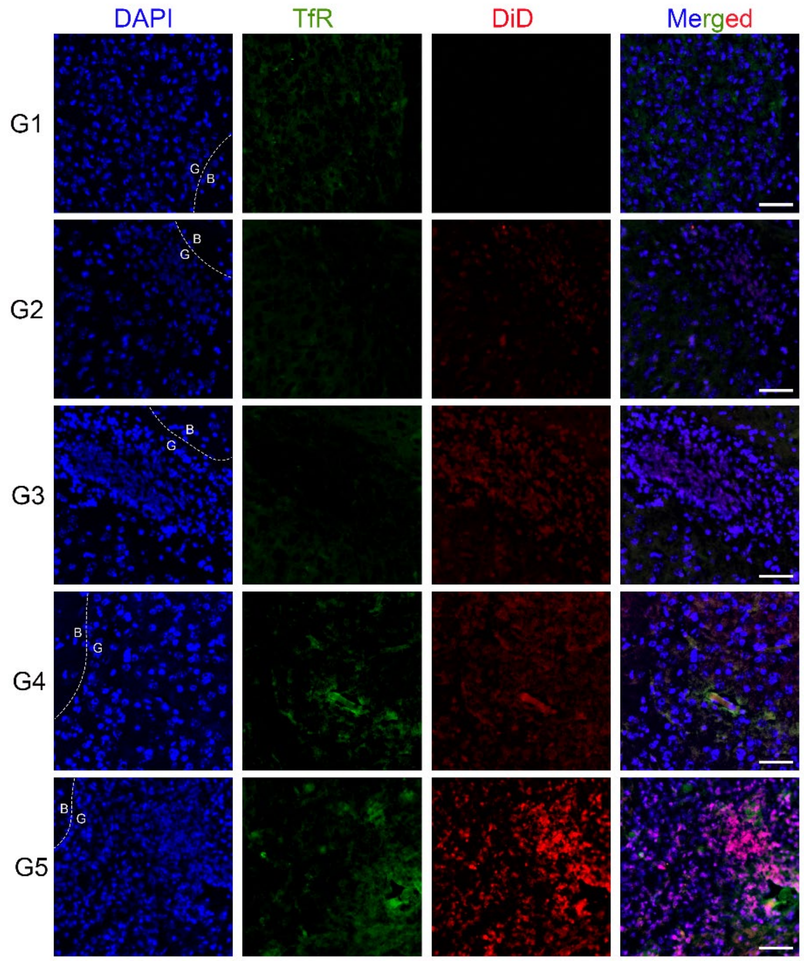

Figure S15. Fluorescence distribution of different exosomes at GBM site after intravenous administration for 24 h, glioma slices were immune-stained with anti-TfR antibody. Scale bar is 50 μm. G1-G5 represents PBS, Exo, PE, ANTE and ACTE, respectively.

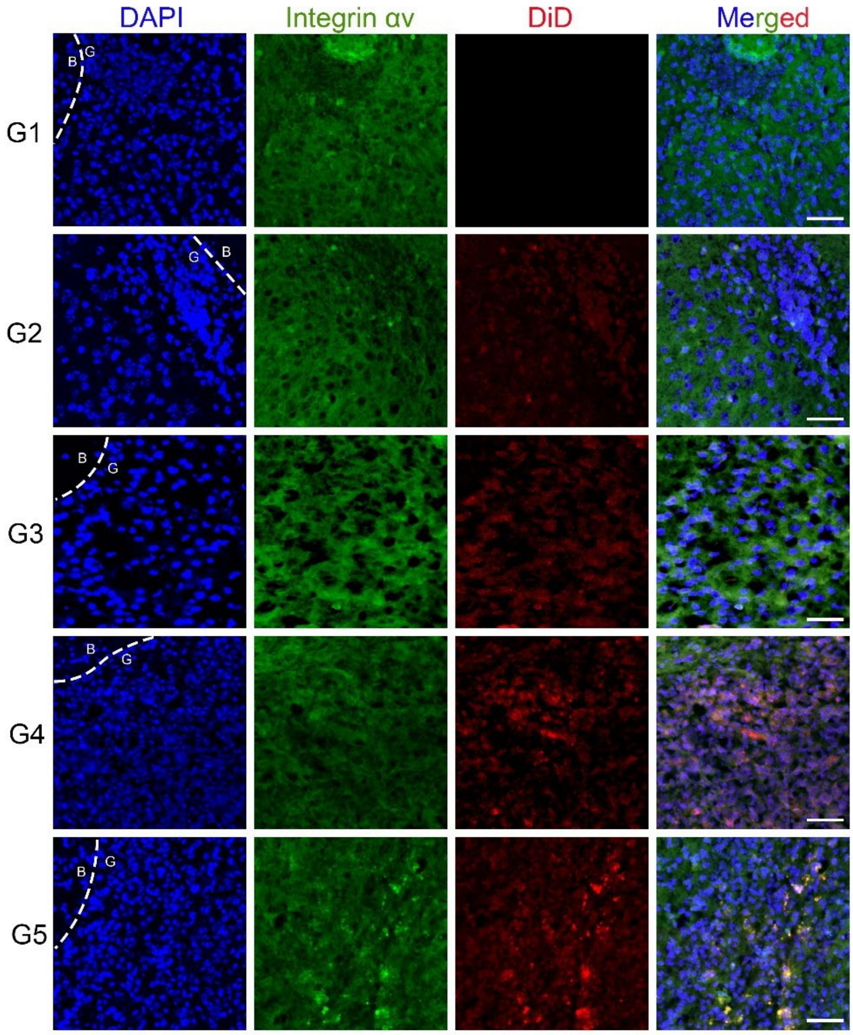

Figure S16. Fluorescence distribution of different exosomes at GBM site after intravenous administration for 24 h, glioma slices were immune-stained with anti-integrin αv antibody. Scale bar is 50 μm. G1-G5 represents PBS, Exo, PE, ANTE and ACTE, respectively.

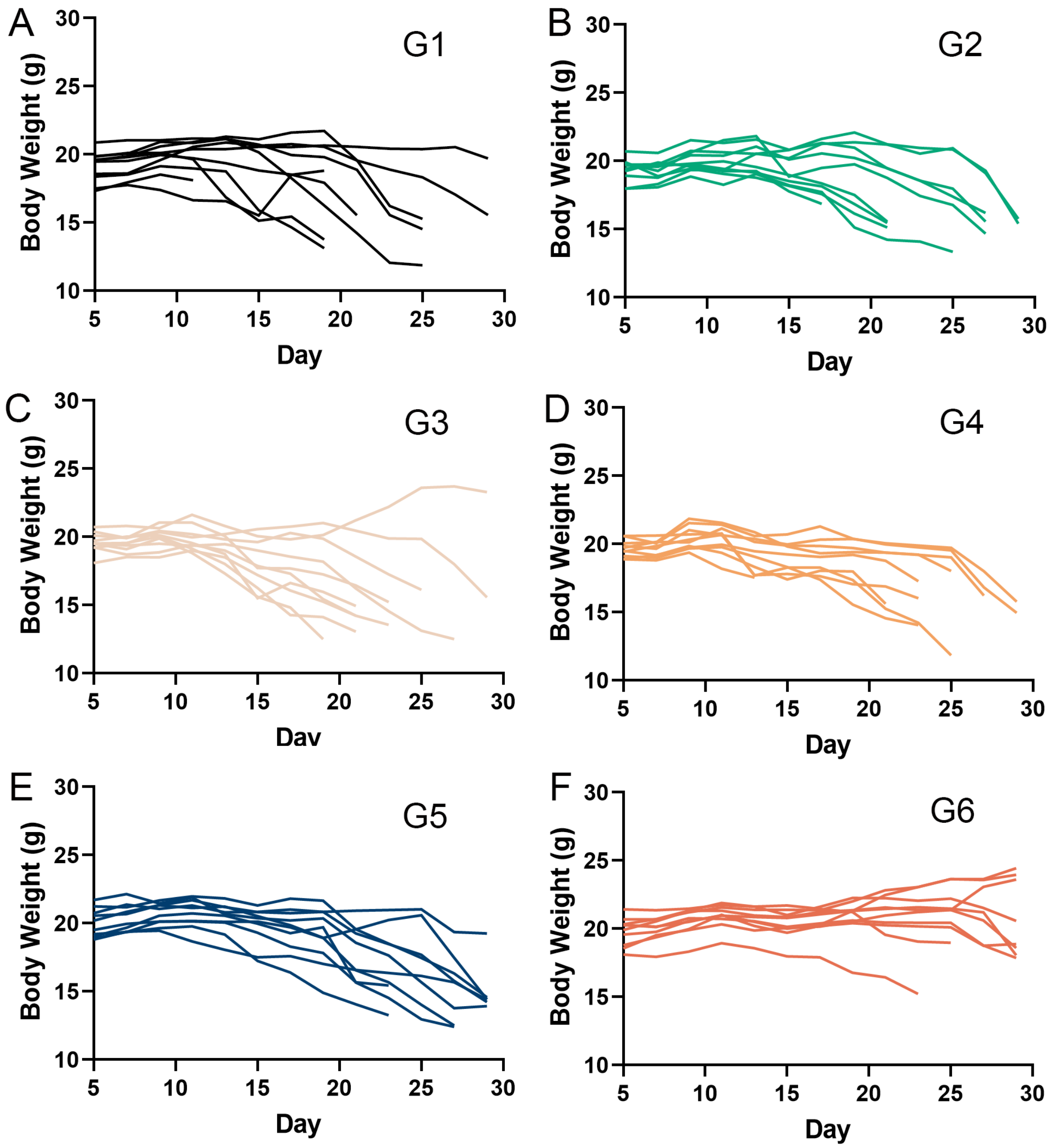

Figure S17. Body weight monitoring of mice after treatment with different formulations. G1-G6 represent PBS, DOX&siTGF-β, Ds@Exo, Ds@PE, Ds@ANTE and Ds@ACTE, respectively.

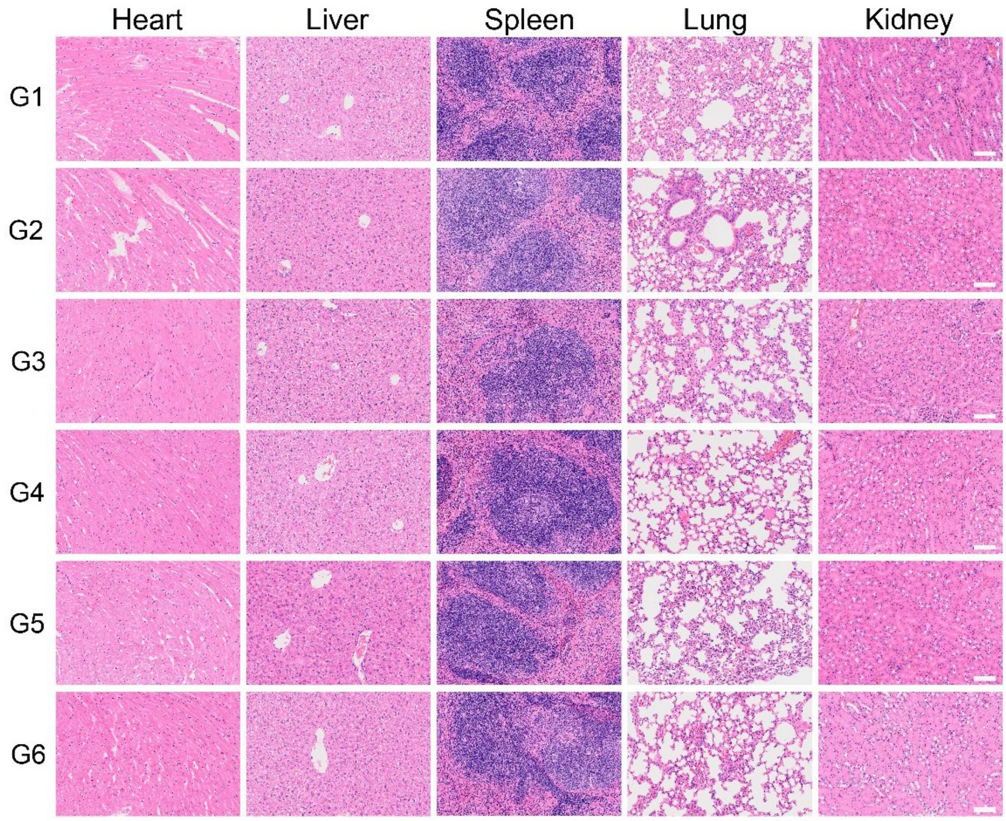

Figure S18. H&E staining of major organs collected at the day after last administration with different formulations. Scale bar is 100 μm. G1-G6 represent PBS, DOX&siTGF-β, Ds@Exo, Ds@PE, Ds@ANTE and Ds@ACTE, respectively.

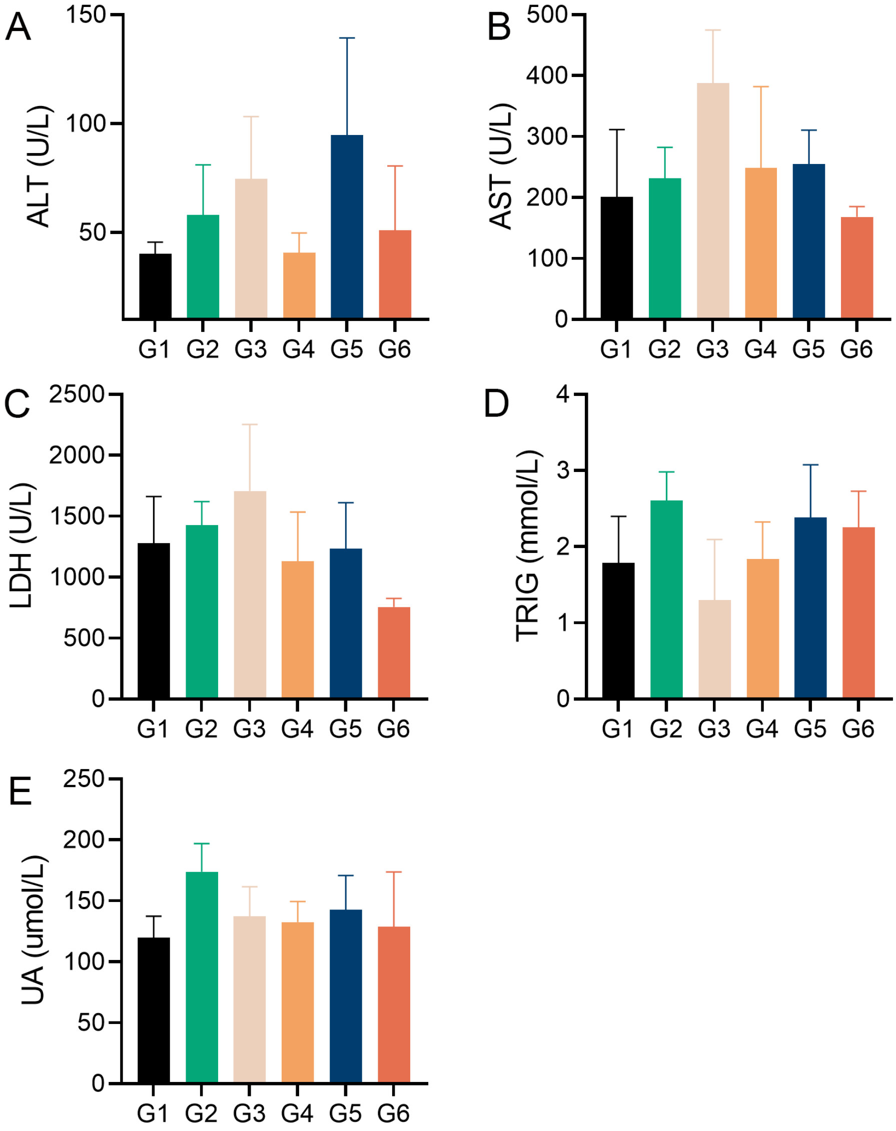

Figure S19. Blood biochemistry analysis of C57BL/6 mice after administration with different formulations (n = 3). (A) alanine transaminase (ALT), (B) aspartate transaminase (AST), (C) lactic dehydrogenase (LDH), (D) Triglyceride (TRIG), (E) uric acid (UA).

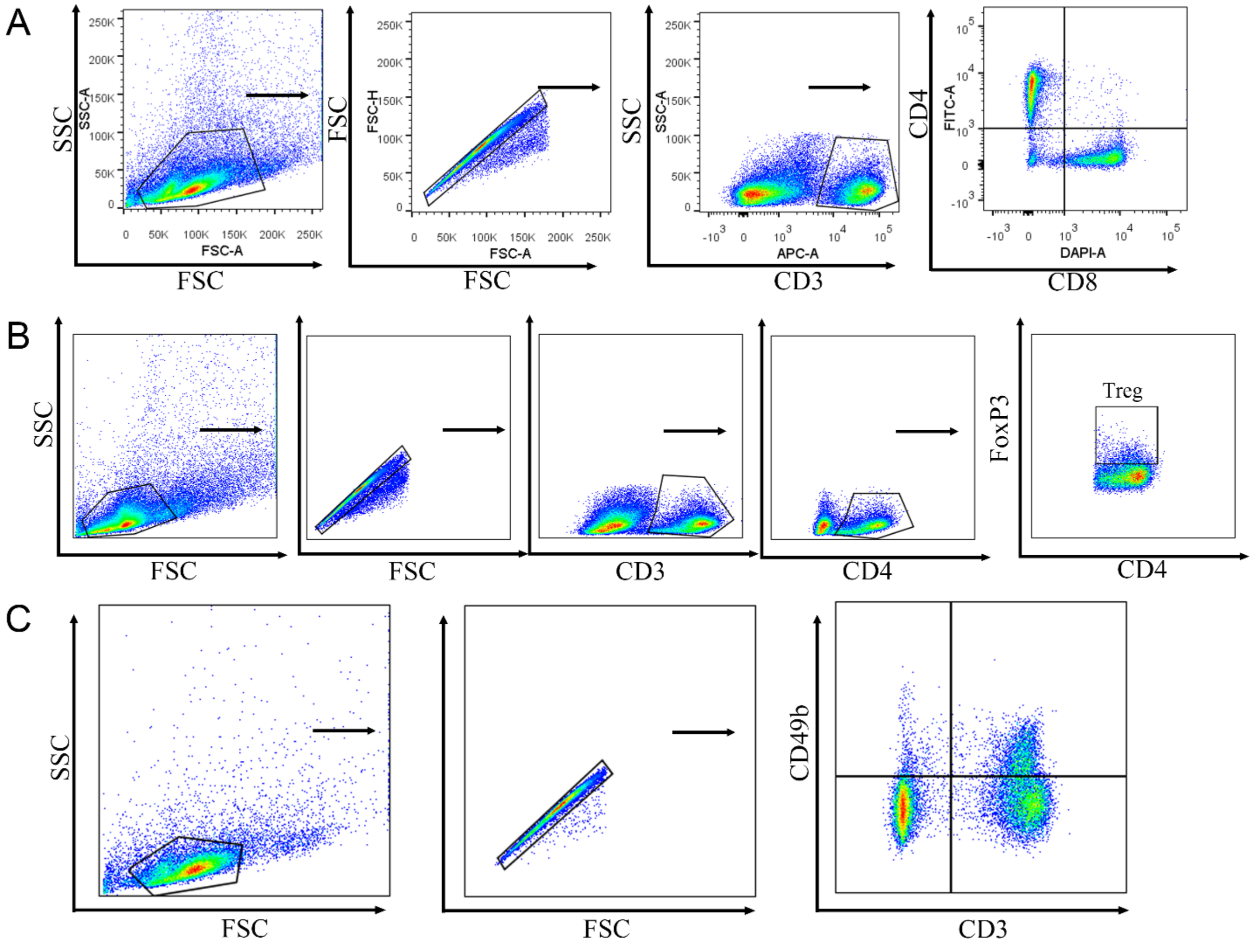

Figure S20. (A) Gating strategy used for flow cytometry analysis of CD3$^+$ lymphocytes, T helper cells (CD3$^+$CD4$^+$) and cytotoxic T cells (CD3$^+$CD8$^+$) in spleen after treatment. (B) Gating strategy used for flow cytometry analysis of CD3$^+$ lymphocytes, Treg cells (CD4$^+$FoxP3$^+$) in spleen after treatment. (C) Gating strategy used for flow cytometry analysis of CD3$^+$ lymphocytes, NK cells (CD3$^-$CD49b$^+$) in DLNs after treatment.
